# Solution phase synthesis of short oligoribonucleotides on a precipitative tetrapodal support

**DOI:** 10.3762/bjoc.10.237

**Published:** 2014-09-29

**Authors:** Alejandro Gimenez Molina, Amit M Jabgunde, Pasi Virta, Harri Lönnberg

**Affiliations:** 1 Department of Chemistry, Faculty of Mathematics and Natural Sciences, University of Turku, FI-20014, Turku, Finland

**Keywords:** nucleic acids, oligoribonucleotides, phosphoramidite, soluble support, synthesis

## Abstract

An effective method for the synthesis of short oligoribonucleotides in solution has been elaborated. Novel 2'-*O*-(2-cyanoethyl)-5'-*O*-(1-methoxy-1-methylethyl) protected ribonucleoside 3'-phosphoramidites have been prepared and their usefulness as building blocks in RNA synthesis on a soluble support has been demonstrated. As a proof of concept, a pentameric oligoribonucleotide, 3'-UUGCA-5', has been prepared on a precipitative tetrapodal tetrakis(4-azidomethylphenyl)pentaerythritol support. The 3'-terminal nucleoside was coupled to the support as a 3'-*O*-(4-pentynoyl) derivative by Cu(I) promoted 1,3-dipolar cycloaddition. Couplings were carried out with 1.5 equiv of the building block. In each coupling cycle, the small molecular reagents and byproducts were removed by two quantitative precipitations from MeOH, one after oxidation and the second after the 5'-deprotection. After completion of the chain assembly, treatment with triethylamine, ammonia and TBAF released the pentamer in high yields.

## Introduction

Recognition of short noncoding RNAs as regulatory elements of gene expression [1–5] has attracted interest in their physico-chemical properties, including structure, dynamics of chain invasion and recognition by small molecular entities [6–10]. The quantities of short RNA sequences required for such studies are often larger than what can easily be obtained by lab-scale solid phase synthesis. In other words, there seems to be a need for a straightforward solution phase approach, allowing assembly of short RNAs in a hundreds of milligrams scale. While several such methods for the synthesis of DNA, based either the phosphoramidite [11–16], H-phosphonate [17–19] or phosphotriester chemistry [20–22], have been introduced, none of them has so far been applied to the synthesis of RNA.

Tetrakis(4-azidomethylphenyl)pentaerythritol has recently been introduced as a practical soluble support for the synthesis of short oligodeoxyribonucleotides [16]. The 3'-terminal nucleoside is attached as a 3'-*O*-(4-pentynoyl) derivative to the support by a Cu(I) catalyzed click reaction and conventional phosphoramidite chemistry is then applied. The advantage of this support is that the small molecular reagents and byproducts are removed in each coupling cycle by two fully quantitative precipitations from MeOH, one after oxidation and the second after the 5'-deprotection. Owing to the symmetrical tetrapodal structure of the support, the completeness of couplings may be verified by ^1^H NMR spectroscopy. We now report on the synthesis of short oligoribonucleotides on this support. Unfortunately, commercially available building blocks that bear a large hydrophobic protecting group at 2'**-**O, such as the *tert*-butyldimethylsilyl group, cannot be used, since the increased hydrophobicity of the growing chain prevents precipitation from MeOH. For this reason, 3'-phosphoramidite building blocks bearing the less hydrophobic 2-cyanoethyl group at 2'-O [23] and the 1-methoxy-1-methylethyl group at 5'**-**O [15] have been prepared and used to assemble a pentamer, 5'-UUGCA-3', on the precipitative pentaerythritol derived support. Precipitation has previously been exploited in conversion of fully protected guanosine 3'-phosphoramidite to cyclic 3',5'-GMP dime by an essentially one-pot synthesis followed by a two-step, one-flask deprotection [24].

## Results and Discussion

### Preparation of nucleosidic building blocks

**Scheme 1:**
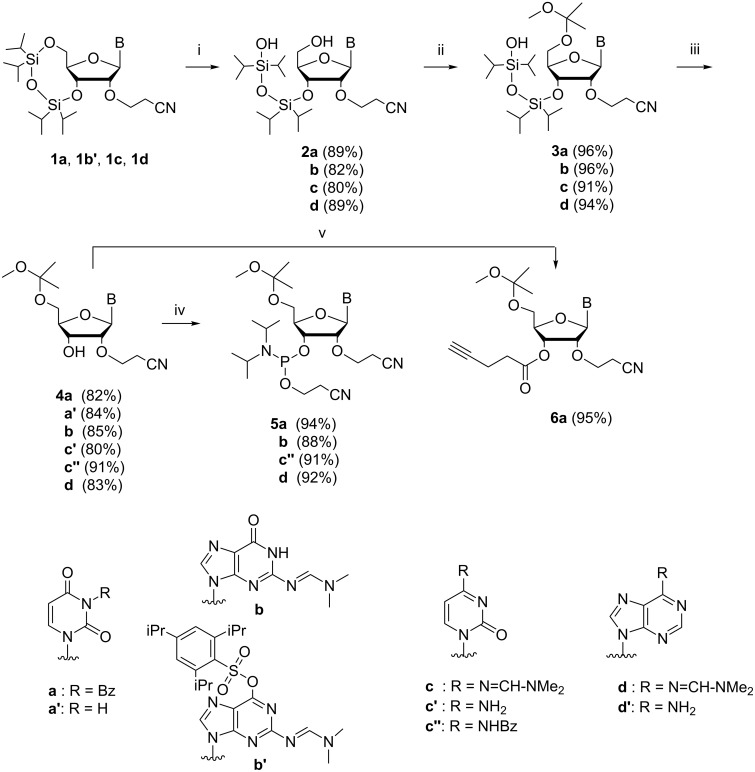
Synthesis of building blocks of the oligoribonucleotide synthesis. (i) TFA, aq THF, 0 °C; (ii) 2-methoxypropene, TsOH, THF; (iii) NH_4_F, MeOH; (iv) 1-chloro-1-(2-cyanoethoxy)-*N*,*N*-diisopropylphosphinamine, DIPEA, DCM; (v) 1. 4-pentynoic acid, DCC, dioxane, 2. Py, DMAP (cat).

The synthesis of the nucleosidic building blocks is outlined in Scheme 1. The base moiety protected 2'-*O*-(2-cyanoethyl)-3',5'-*O*-(1,1,3,3-tetraisopropyldisiloxane-1,3-diyl) ribonucleosides (**1a**,**b'**,**c**,**d**), used as starting materials, were prepared as described previously in the literature [23]. The 5'-O–Si bond was selectively hydrolyzed with TFA in aqueous THF, leaving the 3'-O–Si linkage intact, and the exposed 5'-OH of compounds **2a**–**d** was subjected to acid-catalyzed acetalization with 2-methoxypropene in THF. The subsequent 3'-*O* desilylation of the fully protected nucleosides (**3a**–**d**) then required careful adjustment of conditions. Desilylation with NH_4_F in MeOH turned out to be successful in the sense that the 2'-O and 5'-O protecting groups remained intact, whereas TEA∙3HF in THF partly removed the 5'-*O*-(1-methoxy-1-methylethyl) group and TBAF in THF the 2'-*O*-(2-cyanoethyl) group. Unfortunately, even the reaction with NH_4_F was in some cases accompanied by removal of the base moiety protecting groups. Cytidine derivative **3c** lost entirely and adenosine derivative **3d** partially the dimethylaminomethylene group, yielding **4c'** and **4d'**, respectively. Reintroduction of the same protecting group to **4d'** gave the desired **4d** in high yield, whereas **4c'** was benzoylated to obtain **4c''**. In addition, the uridine derivative **3a** underwent partial removal of the 3-benzoyl group from **3a** giving **4a'** in addition to **4a**. The phosphoramidite building blocks (**5a'**,**b**,**c''**,**d**) were finally obtained by phosphitylation of compounds **4a'**, **4b**, **4c''** and **4d** with 1-chloro-1-(2-cyanoethoxy)-*N*,*N*-diisopropylphosphinamine in MeCN in the presence of *N*,*N*-diisopropylethylamine.

To enable immobilization of the 3'-terminal nucleoside to the azido functionalized support, 3-benzoyl-2-*O*-cyanoethyl-5-(1-methoxy-1-methylethyl)uridine (**4a**) was transformed to its 3'-*O*-(4-pentynoyl) derivative **6a**. To accomplish this, 4-pentynoic acid was first converted to its anhydride by DCC activation in dioxane and this was then used for the 3'-*O*-acylation of **4a** in pyridine in the presence of 4-dimethylaminopyridine. Experimental details for the preparation of all the building blocks and NMR and MS data for their characterization are given in Supporting Information File 1.

### Oligonucleotide synthesis

**Scheme 2:**
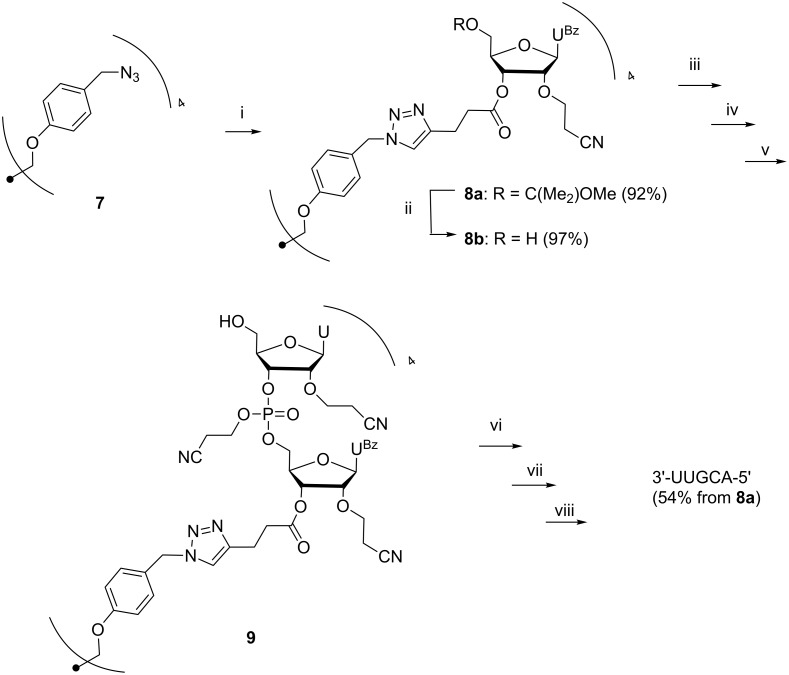
(i) **6a**, CuI, sodium ascorbate, DMAc; (ii) HCl in dioxane/MeOH 2:1, (iii) **5a'**, 4,5-dicyanoimidazole, MeCN/DMF 1:1; (iv) 1. I_2_, THF, 2,6-lutidine, H_2_O, 2. P(OMe)_3_, 3. Precipitation from MeOH; (v) 1. HCl in DCM/MeOH 5:2, 2. Precipitation from MeOH; (vi) steps iii–v repeated to coupled **5b**, **5c''** and **5d** in this order; (vii) 1. TEA, 2. NH_4_OAc, aq NH_3_ (25%), 3. TBAF, iPrNH_2_, THF; (viii) Precipitation from EtOH with NaOAc.

Previously reported [16] pentaerythritol-derived tetraazido support **7** was used to assemble a pentameric oligoribonucleotide, 5'-UUGCA-3', from the building blocks described above. The 3'-terminal block **6a** was first attached to the support by Cu(I) promoted 1,3-dipolar cycloaddition [25–26] (Scheme 2). The procedure employed was essentially the same as described for the attachment of similar 3'-*O-*(4-pentynoyl) acylated 2'-deoxyribonucleosides [16,22]. The tetrapodal nucleoside cluster (**8a**) obtained was purified by silica gel chromatography and the 5'-*O*-(1-methoxy-1-methylethyl) protecting groups were then removed by acid-catalyzed methanolysis with HCl in a mixture of MeOH and dioxane. Removal of volatiles under reduced pressure and treatment of the residue with diethyl ether gave the deprotected support (**8b**) as a white powder. The homogeneity of **8b** was verified by RP-HPLC (see Supporting Information File 1).

**Figure 1:**
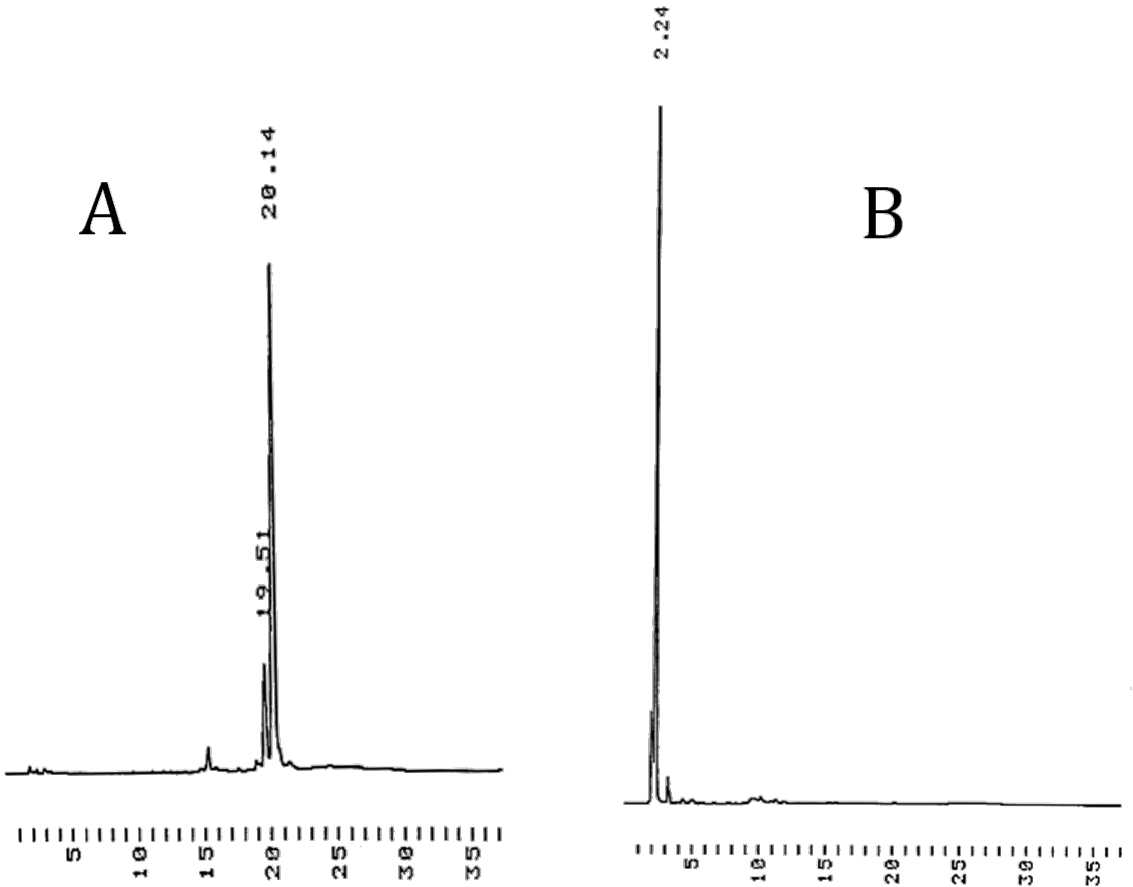
HPLC traces referring to precipitation of the tetrapodal support bearing fully protected UU-dimers (**9**), i.e., after the oxidation step: (A) stands for the precipitate and (B) for the filtrate after the precipitation. Chromatographic conditions: a Thermo ODS Hypersil C18 (250 × 4.6 mm, 5 µm) column eluted with a mixture of MeCN and aq Et_3_N (0.1 mol L^−1^) at flow rate 1 mL min^−1^. A linear gradient from MeCN 25% at *t* = 0 min to MeCN 100% at *t* = 25 min.

The synthetic cycle was composed of three steps, as usual: coupling, oxidation and deprotection. Couplings were carried out in a 1:1 mixture of MeCN and DMF under N_2_ using 1.5 equiv of the respective phosphoramidite building block per a support-bound 5'-hydroxy group. After coupling, the phosphite triester obtained was oxidized to phosphate triester with iodine in aq THF containing 2,6-lutidine, and the reaction was quenched with P(OMe)_3_ in DMF. The mixture was concentrated to oil and the support was precipitated from cold MeOH. HPLC analyses of both the filtrate and the precipitate indicated that the support-bound oligonucleotides precipitated quantitatively from MeOH, while the unreacted building block and small molecular reagents remained in the filtrate (Figure 1). No sign of the HPLC signal referring to the support bound nucleotides (*t*_R_ = 20.14 min) could be detected in the liquid phase. In addition to the signal at *t*_R_ = 20.14 min, a minor signal at *t*_R_ = 19.51 min appears in the HPLC trace of the precipitate. This refers to a compound having lost one benzoyl protecting group.

**Figure 2:**
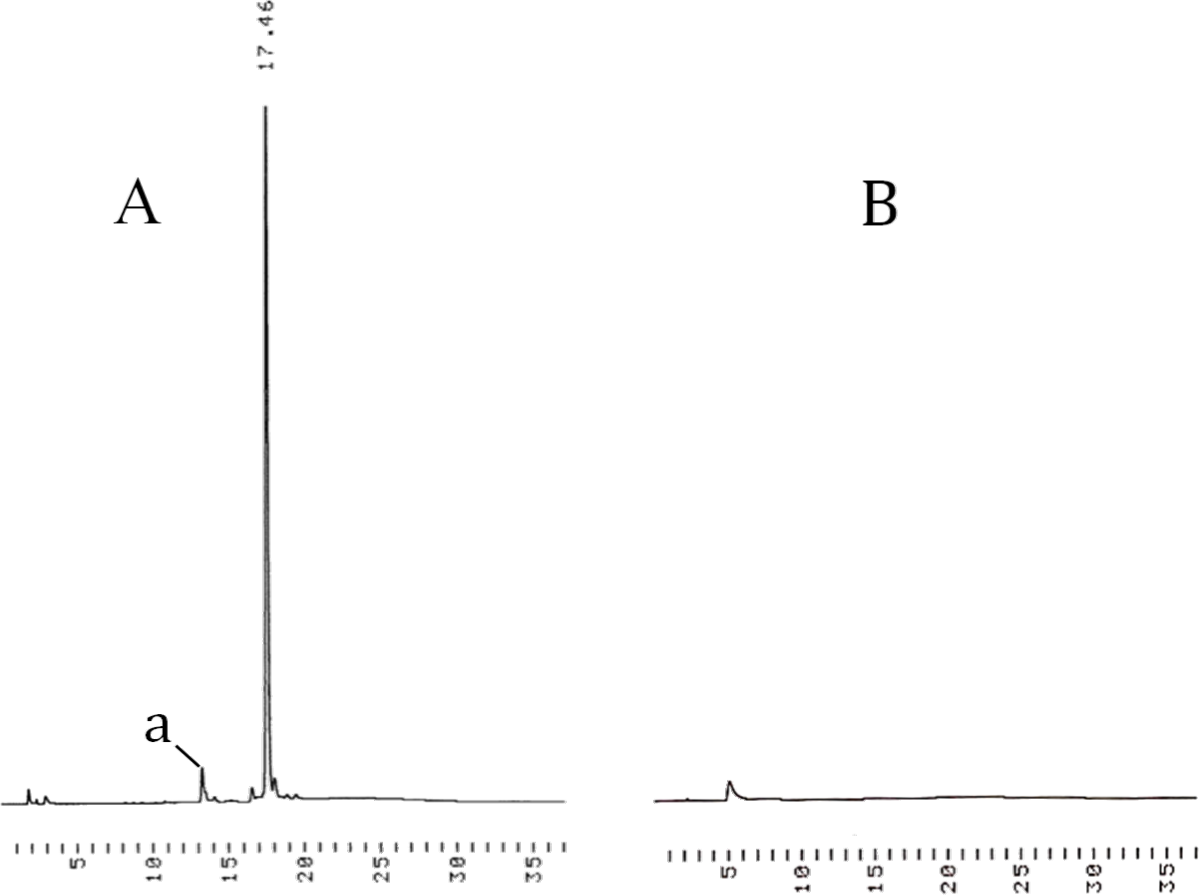
HPLC traces referring to precipitation of the tetrapodal support bearing 5'-*O*-deprotected UU-dimers, i.e., after the deprotection step: (A) stands for the precipitate and (B) for the filtrate after the precipitation. For the chromatographic conditions, see the legend of Figure 1.

The white precipitate was then disolved in a 5:2 mixture (v/v) of DCM and MeOH and subjected to HCl-catalyzed methanolysis ([HCl] = 0.015 mol L^−1^). After 15 min, the mixture was neutralized by the addition of pyridine, concentrated to oil and the support was again precipitated from cold MeOH (Figure 2). The minor signal at 13 min (a) on the HPLC trace of the precipitate (A) refers to a support having one 2-cyanoethyl group removed.

The identity of the product was after each coupling cycle, i.e., after removal of the 5'-O-(methoxy-1-methylethyl) protecting group, verified by ESIMS. The data obtained are given in the Table 1. The completeness of the coupling was verified by HPLC. To ensure quantitative coupling, an exceptionally long coupling time (12 h) was employed. The coupling time was not really optimized, but preliminary studies revealed that after 4 h a minor part of the support still contained one unreacted branch. Since 4,5-dicyanoimidazole afforded almost quantitative coupling, no other activators were tested.

**Table 1:** Negative ion ESIMS of the tetrapodal pentaerythritol (PE) soluble support bearing 5'-*O*-deprotected oligonucleotides.

Compound	*m*/*z* obsd.	*m*/*z* calcd

PE-(U^Bz^)_4_	1327.8 [M + 2Cl]^2−^	1327.7 [M + 2Cl]^2−^
PE-[U^Bz^U]_4_	2116.4 [M − 2H]^2−^	2116.8 [M − 2H]^2−^
PE[U^Bz^UG^dmf^]_4_	3130.8 [M − 2H]^2−^	3128.6 [M − 2H]^2−^
PE[U^Bz^UG^dmf^C^Bz^]_4_	2079.5 [M − 4H]^4−^	2079.7 [M − 4H]^4−^
PE[U^Bz^UG^dmf^C^Bz^A^dmf^]_4_	2570.0 [M − 4H]^4−^	2570.1 [M − 4H]^4−^

**Figure 3:**
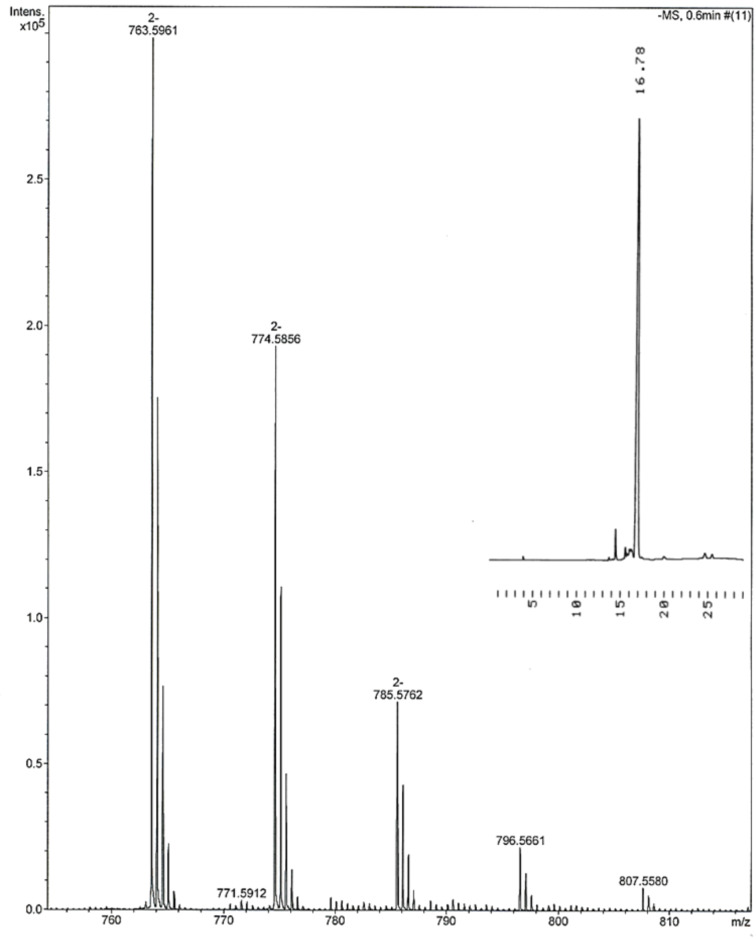
Negative ion ESIMS and HPLC traces of the isolated 3'-UUGCA-5'. For the MS over a wider mass range, see Supporting Information File 1. Chromatographic conditions: a Thermo ODS Hypersil C18 (250 × 4.6 mm, 5 µm) column eluted with a mixture of MeCN and H_2_O at flow rate 1 mL min^−1^. A linear gradient from MeCN 0% at *t* = 0 min to MeCN 100% at *t* = 20 min. The minor signal at 14.5 min refers to benzamide.

Upon completion of the assembly of 3'-UUGCA-5', the oligonucleotide was released from the support and deprotected by treatment with triethylamine, ammonia and finally with TBAF. The deprotected pentamer was precipitated from EtOH with NaOAc, The overall yield of the pentamer was 54% (according to the UV absorption), corresponding to 86% average coupling yield. Figure 3 shows the homogeinety and identity of the pentamer.

## Conclusion

An effective method for the synthesis of short oligoribonucleotides in solution has been elaborated. The main characteristics of this method are the use of a precipitative pentaerythritol-derived soluble support and small reasonably polar sugar moiety protecting groups, viz. the 2'-*O*-(2-cyanoethyl) and the 5'-*O*-(1-methoxy-1-methylethyl) group. These together allow facile purification after each coupling/oxidation and 5**'**-*O*-deprotection step by quantitative precipitation from MeOH. As a proof of concept, a 3'-UUGCA-5' pentamer was prepared in 54% yield by using 1.5 equiv of the monomeric phosphoramidite building block per 5'-hydroxy function. Release from the support and removal of the cyanoethyl group from the phosphate and the base moiety protecting groups was achieved by consecutive treatments with triethylamine and aqueous NH_3_/NH_4_OAc. Treatment of the released oligomer with 1 mol L^−1^ TBAF in THF removed the 2'-*O*-(2-cyanoethyl) protecting group and the deprotected oligomer was isolated by precipitation from EtOH with NaOAc.

## Experimental

**General**. RP-HPLC separations were carried out on a Thermo ODS Hypersil C18 (250 × 4.6 mm, 5 µm) analytical column using UV detection (λ = 260 nm). NMR spectra were recorded on a Bruker Avance spectrometer (500 or 400 MHz) at 25 °C. Chemical shifts are given in ppm from TMS. HRMS analysis were recorded on a Bruker Daltonics spectrometer.

For the preparation and characterization of building blocks **1a**,**b'**,**c**,**d**, **2a**–**d**, **3a**–**d**, **4a**,**a'**,**b**,**c'**,**d**, **5a'**,**b**,**c''**,**d** and **6a**, see the Supporting Information File 1.

**Tetrakis-*O*-(4-azidomethylphenyl)pentaerythritol** (**7**). Preparation of compound **7** has been described previously [16].

**Tetrakis-*O*-{4-{3-[3-benzoyl-2'-*O*-(2-cyanoethyl)-5'-*O*-(1-methoxy-1-methylethyl)uridin-3'-*O*-yl]-3-oxoprop-1-yl}-1*H*-1,2,3-triazol-1-ylmethylphenyl}pentaerythritol** (**8a**). Compounds **6a** (0.544 g; 0.98 mmol) and **7** (0.130 g; 0.20 mmol) were dissolved in dry DMAc (5 mL) in a pyrex tube. The slightly yellow-brownish solution was subjected to degassing according to a previous procedure [27]. A catalytic amount of CuI and sodium ascorbate were added under N_2_ and the mixture was stirred for 20 h at 45 °C. The solvent was removed under reduced pressure and the residue was subjected to column chromatography on silica gel using a gradient of 1–5% MeOH in DCM containing 1% TEA. Support **8a** was obtained as a white foam in 92% yield (0.520 g; 0.18 mmol).

**Tetrakis-*O*-{4-{3-[3-benzoyl-2'-*O*-(2-cyanoethyl)uridin-3'-*O*-yl]-3-oxoprop-1-yl}-1*H*-1,2,3-triazol-1-ylmethylphenyl}pentaerythritol** (**8b**). Support **8a** (0.520 g; 0.18 mmol) was dissolved in 2:1 mixture (v/v) of dioxane and MeOH (20 mL) and 0.5 mL of 0.1 mol L^−1^ HCl in dioxane was added. The yellowish solution was stirred for 1 h at room temperature and the solvent removed under reduced pressure. A small amount of pyridine was added to neutralize any traces of acid. Et_2_O (50 mL) was added affording **8b** instantaneously as white precipitate (0.453 g; 0.17 mmol) in 97% yield. The identity of the product was verified by ESIMS (Table 1) and the homogeinity by HPLC on a Thermo ODS Hypersil C18 (250 × 4.6 mm, 5 µm) column eluted with a mixture of MeCN and H_2_O at flow rate 1 mL min^−1^, using a linear gradient from MeCN 25% at *t* = 0 min to MeCN 100% at *t* = 25min (for the HPLC traces, see Supporting Information File 1).

**Oligonucleotide synthesis**. Support **8b** (0.151 g; 0.056 mmol) and 6 equiv of the phosphoramidite building block **5a'** (200 mg; 0.35 mmol; 1.5 equiv per branch) were dissolved in an 1:1 mixture (v/v) of MeCN and DMF (5 mL), and 4,5-dicyanoimidazole in MeCN (1.39 mL of 0.25 mol L^−1^ solution, 0.32 mmol) was added. The mixture was stirred for 12 h at room temperature and then I_2_ (0.13 g; 0.5 mmol) in 4:2:1 mixture (v/v) of THF, water and 2,6-lutidine (4 mL) was added and the mixture was stirred for 20 min. Unreacted I_2_ was quenched by the addition of P(OMe)_3_ (0.1 mmol), the mixture was concentrated to oil and the oligonucleotide was precipitated in quantitative yields from cold MeOH (75 mL). The white precipitate was filtered over celite (AW) and isolated.

The white precipitate obtained was dissolved in a 5:2 mixture of DCM and MeOH (35 mL) and 5 mL of a 0.125 mol L^−1^ HCl in MeOH solution were added. After 15 min, pyridine was added to neutralize the acid catalyst. The solution was concentrated to oil and cold MeOH (50 mL) was added. The white precipitate obtained instantaneously was collected by filtration through celite (AW) and dried under vacuum. The identitiy of the clustered dimer PE[3'-U^Bz^U-5'-OH]_4_ (**9**) was verified by ESIMS (Table 1) and the homogeneity by HPLC (Figure 2). The gravimetrically determined overall yield was 93% (230 mg).

The coupling cycle (coupling, oxidation, precipitation, deprotection and precipitation) was then repeated to couple building blocks **5b**, **5c''** and **5d**, in this order. The identity and homogeneity of the product was verified after each cycle by ESIMS (Table 1) and HPLC.

**Release and deprotection**. A sample of the support bound cluster, 3'-U^Bz^UG^dmf^C^Bz^A^dmf^-5' (20 mg), was dissolved in an 1:1 mixture of DMF and MeCN (4 mL) and treated with TEA (2 mL) to release the protected oligonucleotide from the support [23]. To the mixture, aq NH_3_ (25%) containing 10% NH_4_OAc (w/v) was added and the solution was stirred for 12 h at room temperature [28]. The solvent was removed under reduced pressure and 1 mol L^−1^ solution of TBAF in dry THF (4 mL) and isopropylamine (200 µL) were added. The reaction was monitored by HPLC and was observed to be completed in 12 h at room temperature. The homogeneity and identity of the deprotected pentamer, 3'-UUGCA-5', was verified by HPLC and ESIMS (Figure 3). According to the UV-spectrophotometric assay, the overall yield of the pentamer from **8b** was 54%, corresponding to 86% average coupling yield. The remaining support bound oligonucleotide was then released and deprotected in the same manner, and the deprotected oligonucleotide was precipitated as a sodium salt by adding EtOH (20 mL) to the solution of the oligonucleotide in 3 mol L^−1^ aq NaOAc (1.0 mL) and keeping the mixture at −20 °C for 1.5 h. The precipitate was carefully dried, and the concentration of a weight sample dissolved in water was determined on the basis of UV absorbance at 260 nm. According to this analysis, the oligonucleotide content of the precipitate was 95%.

## Supporting Information

File 1 Further experimental data.
