# Supplementary material for: Solution phase synthesis of short oligoribonucleotides on a precipitative tetrapodal support
Source: Beilstein J Org Chem. 2014 Sep 29;10:2279–85. doi: 10.3762/bjoc.10.237 (PMC4187100; doi:10.3762/bjoc.10.237)
Supplement: File 1 — Further experimental data. [file Beilstein_J_Org_Chem-10-2279-s001.pdf]

Supporting Information

for

Solution phase synthesis of short oligoribonucleotides on a precipitative tetrapodal support

Alejandro Gimenez Molina, Amit M. Jabgunde, Pasi Virta and Harri Lönnberg*

Address: Department of Chemistry, Faculty of Mathematics and Natural Sciences, University of Turku, FI-20014, Turku, Finland

Email: Harri Lönnberg - harlon@utu.fi

*Corresponding author

Further experimental data

Content

Synthesis of **1a–d** ---------- S3

Synthesis of **2a**,**b** ---------- S3

Synthesis of **2c**,**d** ---------- S4

Synthesis of **3a–c** ---------- S5

Synthesis of **3d** ---------- S6

Synthesis of **4a**,**a´** ---------- S6

Synthesis of **4b**, **4c´** and **4c´´** ---------- S7

Synthesis of **4d** ---------- S8

Synthesis of **5a´**,**b** ---------- S8

Synthesis of **5c´´**, **5d** ---------- S9

Synthesis of **6a** ---------- S9

^{1}H and ^{13}C NMR spectrum of **2a** ---------- S11

^{1}H and ^{13}C NMR spectrum of **2b** --- S12
^{1}H and ^{13}C NMR spectrum of **2c** --- S13
^{1}H and ^{13}C NMR spectrum of **2d** --- S14
^{1}H and ^{13}C NMR spectrum of **3a** --- S15
^{1}H and ^{13}C NMR spectrum of **3b** --- S16
^{1}H and ^{13}C NMR spectrum of **3c** --- S17
^{1}H and ^{13}C NMR spectrum of **3d** --- S18
^{1}H and ^{13}C NMR spectrum of **4a** ------------------------------- --- S19
^{1}H and ^{13}C NMR spectrum of **4a’** -- S20
^{1}H and ^{13}C NMR spectrum of **4b** ------------------------ --- S21
^{1}H and ^{13}C NMR spectrum of **4c’** -- S22
^{1}H and ^{13}C NMR spectrum of **4c’’** --- S23
^{1}H and ^{13}C NMR spectrum of **4d** --- S24
^{31}P NMR spectrum of **5a’** and **5b**--- S25
^{31}P NMR spectrum of **5c’’ -**-- S26
^{31}P NMR spectrum of **5d -**-- S26
^{1}H and ^{13}C NMR spectrum of **6a -**--- S27
RP-HPLC traces for **8b**-- S28
ESI-MS of 3´-UUGCA-5´-- S29

3-Benzoyl-2′-*O*-(2-cyanoethyl)-3′,5′-*O*-(1,1,3,3-tetraisopropyldisiloxane-1,3-diyl)uridine (**1a**) was prepared as described in literature [1]. The ^{1}H and ^{13}C NMR spectra were identical with those reported earlier [1]. Positive ion ESI-MS: *m/z* obsd. 644.30 $[M+H]^+$, 666.28 $[M+Na]^+$; calcd. 644.28 $[M+H]^+$, 666.26 $[M+Na]^+$

2′-*O*-(2-Cyanoethyl)-*N*^{2}-(dimethylaminomethylene)-3′,5′-(1,1,3,3-tetraisopropyldisiloxane-1,3-diyl)guanosine (**1b**) was prepared as described in literature [1]. The ^{1}H and ^{13}C NMR spectra were identical with those reported earlier [1]. Positive ion ESI-MS: *m/z* obsd. 900.44 $[M+H]^+$; calcd. 900.45 $[M+H]^+$

2′-*O*-(2-Cyanoethyl)-*N*^{4}-(dimethylaminomethylene)-3′,5′-(1,1,3,3-teraisopropyldisiloxane-1,3-diyl)cytidine (**1c**) was prepared as described in literature [1]. The ^{1}H and ^{13}C NMR spectra were identical with those reported earlier [1]. Positive ion ESI-MS: *m/z* obsd. 594.34 $[M+H]^+$; calcd. 594.31 $[M+H]^+$.

2′-*O*-(2-Cyanoethyl)-*N*^{6}-(dimethylaminomethylene)-3′,5′-(1,1,3,3-tetraisopropyldisoloxane-1,3-diyl)adenosine (**1d**) was prepared as described in literature [1]. The ^{1}H and ^{13}C NMR spectra were identical with those reported earlier [1]. Positive ion ESI-MS: *m/z* obsd. 618.35 $[M+H]^+$; calcd. 618.33 $[M+H]^+$.

3-Benzoyl-2′-*O*-(2-cyanoethyl)-3′-*O*-(1,1,3,3-tetraisopropyldisiloxane-1-yl)uridine (**2a**). Compound **1a** (2.35g, 3.65mmol) was dissolved in THF (47 mL), 1:1 mixture (*v/v*) of TFA and water (23.5 mL) was added dropwise on an ice-bath and the mixture was left with stirring for 3.5 h at 0 °C. The solution was extracted with EtOAc (150 mL), and the organic phase was washed with aq $NaHCO_3$ (2 x 300 mL) and dried over Na_2SO_4. Purification by column chromatography on silica gel using a gradient of 1-10% MeOH in DCM gave compound **2a** in 89% yield as white foam (2.15 g, 3.25 mmol). ^{1}H-NMR (400 MHz, $CDCl_3$): δ = 0.86-1.20 (m, 28H), 2.61 (t, *J* = 5.9 Hz, 2H), 3.80-4.21 (m, 8H), 4.49-4.52 (m, 1H), 5.76 (d, *J* = 8.2 Hz, 1H), 5.78 (s, 1H), 7.50 (t, *J* = 7.4 Hz, 2H), 7.67 (t, *J* = 7.4 Hz, 1H), 7.91 (d, *J* = 7.4 Hz, 2H), 8.43(d, *J* = 8.2 Hz, 1H). ^{13}C NMR (100 MHz, $CDCl_3$): δ = 12.8, 13.1, 13.4, 13.6, 17.0, 17.1, 17.2, 18.9, 58.6, 64.9, 66.8, 83.0, 83.5, 88.6, 101.0, 117.5, 129.3, 130.5, 131.2, 135.4, 140.7, 149.2, 162.6, 168.5. For the spectra, see page S11. Positive ion ESI-HRMS: *m/z* obsd. 684.2734 $[M+Na]^+$; calcd. 684.2749 $[M+Na]^+$.

2′-*O*-(2-Cyanoethyl)-*N*^{2}-(dimethylaminomethylene)-3′-*O*-(tetraisopropyldisiloxane-1-yl)guanosine (**2b**). Compound **1b** (2.30 g, 3.63 mmol) was dissolved in THF (40 mL) and 1:1 mixture (*v/v*) of TFA and water (6.6 mL) was added dropwise on an ice-bath. The reaction mixture was allowed to stand 3h on the ice-bath and then another portion (6.6 mL) of aq TFA was added. The mixture was stirred for 4.5 h on the ice-bath and extracted with EtOAc (150 mL). The organic phase

was washed with aq. $NaHCO_3$ (2 x 300 mL) and dried over Na_2SO_4. Purification by column chromatography on silica gel using a gradient of 1-10% MeOH in DCM gave compound **2b** in 82% yield as white foam (2.53 g; 2.98 mmol). ^{1}H NMR (400 MHz, $CDCl_3$): δ = 0.95-1.13 (m, 28H), 2.69 (t, *J* = 5.9 Hz, 2H), 3.09 (s, 3H), 3.19 (s, 3H), 3.85-4.16 (m, 4H), 4.22-4.30 (m, 2H), 4.50-4.53 (m, 1H), 6.03 (s, 1H), 7.94 (s, 1H), 8.66 (s, 1H). ^{13}C NMR (100 MHz, $CDCl_3$): δ = 12.6, 12.9, 13.0, 13.4, 16.9, 17.1, 17.3, 17.5, 19.3, 35.2, 41.3, 60.4, 66.4, 69.7, 81.1, 83.7, 87.6, 117.6, 122.2, 135.4, 149.3, 156.9, 157.8, 158.2. For the spectra, see page S12. Positive ion ESI-HRMS: *m/z* obsd. 652.3333 $[M+H]^+$; calcd. 652.3310 $[M+]^+$.

2’-*O*-(2-Cyanoethyl)-*N*⁴-(dimethylaminomethylene)-3’-*O*-(1,1,3,3-tetraisopropyldisiloxane-1-yl)cytidine (**2c**). Compound **1c** (3.10 g, 5.22 mmol) was hydrolyzed to **2c** as described above for the hydrolysis of **1b** to **2b.** Purification by column chromatography on silica gel using a gradient of 1-10% MeOH in DCM gave compound **2c** as white foam (1.31 g; 2.14 mmol). Additionally, 2’-*O*-(2-cyanoethyl)-3’-*O*-(1,1,3,3-tetraisopropyldisiloxane-1-yl)cytidine (1.21 g, 2.17 mmol) was obtained. To introduce the base moiety protection lost during the hydrolysis, the compound was dissolved in dry MeOH (140 mL) and 3.35 equiv. of *N*,*N*-dimethylformamide dimethylacetal (1.03 mL; 7.75 mmol) was added. After 4 h at room temperature, the solvent was removed and **2c** (1.25 g; 2.04 mmol) formed was isolated by column chromatography on silica using a gradient of 1-10% MeOH in DCM. Accordingly, the overall yield of **2c** was 80% (2.56 g; 4.19 mmol). ^{1}H NMR (400 MHz, $CDCl_3$): δ = 0.87-1.12 (m, 28H), 2.72 (t, *J* = 6.4 Hz, 2H), 3.13 (s, 3H), 3.16 (s, 3H), 3.86-4.00 (m, 3H), 4.08-4.15 (m, 2H), 4.22-4.26 (m, 1H), 4.46-4.48 (m, 1H), 5.80 (s, 1H), 6.03 (d, *J* = 7.2 Hz, 1H), 8.27 (d, *J* = 7.2 Hz, 1H), 8.80 (s, 1H). ^{13}C NMR (100 MHz, $CDCl_3$): δ = 12.6, 12.9, 13.0, 13.4, 16.9, 17.1, 17.3, 17.5, 19.0, 35.2, 41.5, 58.8, 65.0, 66.9, 82.7, 83.1, 89.8, 102.4, 117.7, 135.4, 149.3, 156.9, 158.2 158.7. For the spectra, see page S13. Positive ion ESI-HRMS: *m/z* obsd. 612.3259 $[M+H]^+$; calcd. 612.3249 $[M+]^+$.

2’-*O*-(2-Cyanoethyl)-*N*⁴-dimethylaminomethylene-3’-O-(1,1,3,3-tetraisopropyldisiloxane-1-yl)adenosine (**2d**). Compound **1d** (310 mg, 0.502 mmol) was hydrolyzed to **2d** as described above for the hydrolysis of **1b** to **2b**. Purification by column chromatography on silica gel using a gradient of 1-10% MeOH in DCM gave compound **2d** in 89% yield as white foam (284 mg; 0.447 mmol). ^{1}H NMR (400 MHz, $CDCl_3$): δ = 0.97-1.08 (m, 28H), 2.45 (m, 2H), 3.23 (s, 3H), 3.28 (s, 3H), 3.40-3.48 (m, 1H), 3.72-3.35 (m, 3H), 3.96-4.04 (m, 1H), 4.29-4.31 (br s, 1H), 4.76-4.84 (m, 2H), 5.99 (s, 1H), 8.04 (s, 1H), 8.49 (s, 1H), 8.98 (s, 1H); ^{13}C NMR (100 MHz, $CDCl_3$): δ = 13.1, 13.3, 13.4, 13.5, 17.1, 17.2, 17.3, 17.4, 18.8, 35.2, 41.4, 62.4, 65.5, 71.5, 81.4, 88.5, 89.4, 117.0, 127.6, 141.7, 150.0, 152.1, 158.4, 160.4. For the spectra, see page S14. Positive ion ESI-HRMS: *m/z* obsd. 636.3353 $[M+H]^+$; calcd. 636.3361 $[M+]^+$.

3-Benzoyl-2'-*O*-(2-cyanoethyl)-5'-*O*-(1-methoxy-1-methylethyl)-3'-*O*-(1,1,3,3-tetraisopropyl-disiloxane-1-yl)uridine (**3a**). Compound **2a** (2.53 g, 3.88 mmol) was dissolved in dry THF (60 mL) and 10 equiv. of 2-methoxypropene (3.90 mL, 40.7 mmol) was added. A catalytic amount of *p*-toluenesulfonic acid monohydrate (15.0 mg, 0.077 mmol) dissolved in dry THF (0.5 mmol) was added. The progress of acetalization was monitored by TLC and *p*-toluensulfonic acid monohydrate was added portionwise until the starting material had disappeared. The crude mixture was extracted with EtOAc (100 mL). The organic phase was washed with aq. $NaHCO_3$ (200 mL) and dried over Na_2SO_4. Compound **3a** was obtained in 96% yield as white foam (2.70 g, 3.73 mmol). ^{1}H NMR (400 MHz, $CDCl_3$): δ = 0.98-1.19 (m, 28H), 1.56 (s, 3H), 1.66 (s, 3H), 2.79 (t, *J* = 6.1 Hz, 2H), 3.25 (s, 3H), 3.59-3.92 (m, 2H), 3.97-4.07 (m, 2H), 4.20-4.30 (m, 2H), 4.68-4.72 (m, 1H), 5.78 (d, *J* = 8.2 Hz, 1H), 5.79 (s, 1H), 7.58 (t, *J* = 7.4 Hz, 2H), 7.75 (t, *J* = 7.4 Hz, 1H), 8.02 (d, *J* = 7.4 Hz, 2H), 8.36 (d, *J* = 8.2 Hz, 1H. ^{13}C NMR (100 MHz, $CDCl_3$): δ = 13.1, 13.4, 13.8, 14.5, 16.7, 16.9, 17.0, 17.2, 18.4, 23.5, 26.4, 48.1, 57.6, 66.0, 67.7, 81.4, 82.7, 89.2, 99.6, 100.3, 117.9, 129.2, 130.2, 135.0, 140.1, 149.2, 161.9, 169.1. For the spectra, see page S15. Positive ion ESI-HRMS: *m/z* obsd. 756.3299 $[M+Na]^+$; calcd. 756.3324 $[M+Na]^+$.

2'-*O*-(2-Cyanoethyl)-*N*^{2}-(dimethylaminomethylene)-5'-*O*-(1-methoxy-1-methylethyl)-3'-*O*-(1,1,3,3-tetraisopropyldisiloxane-1-yl)guanosine (**3b**). Compound **2b** (2.53 g, 3.88 mmol) was transformed to **3b** as described above for the transformation of **2a** to **3a**. dissolved in dry THF (60 mL) and to the slightly yellowish solution were added 10 equiv of 2-methoxypropene (3.90 mL, 40.7 mmol). Compound **3b** was obtained in 96% yield as white foam (2.70 g, 3.73 mmol). ^{1}H NMR (400 MHz, CDCl3): δ = 0.90-1.08 (m, 28H), 1.40 (s, 6H), 2.65 (dd, *J* = 12.3 and 6.2 Hz, 2H), 3.10 (s, 3H), 3.18 (s, 3H), 3.19 (s, 3H), 3.60-3.68 (m, 2H), 3.70-3.76 (m, 1H), 3.80-3.90 (m, 3H), 4.24-4.33 (m, 2H), 4.72-4.73 (m, 1H), 6.16 (s, 1H), 8.03 (s, 1H), 8.64 (s, 1H). ^{13}C NMR (100 MHz, $CDCl_3$): δ = 13.2, 13.3, 13.4, 13.5, 17.1, 17.2, 17.2, 17.3, 18.9, 24.1, 24.4, 35.2, 41.3, 48.8, 59.5, 65.5, 70.6, 82.9, 83.4, 85.8, 100.6, 117.6, 120.3, 136.1, 149.7, 156.9, 157.6, 158.2. For the spectra, see page S16. Positive ion ESI-HRMS: *m/z* obsd. 724.3885 $[M+H]^+$; calcd. 724.3885 $[M+]^+$.

2'-*O*-(2-Cyanoethyl)-*N*^{4}-(dimethylaminomethylene)-5'-*O*-(1-methoxy-1-methylethyl)-3'-*O*-(1,1,3,3-tetraisopropyldisiloxane-1-yl)cytidine (**3c**). Compound **2c** (2.00 g, 3.27 mmol) was transformed to **3c** as described above for the transformation of **2a** to **3a**. Purification by column chromatography on silica gel using a stepwise gradient of 1-5% MeOH in DCM containing 1% triethylamine gave **3c** in 91% yield as white foam (2.03 g, 2.97 mmol). ^{1}H NMR (400 MHz, $CDCl_3$): δ = 0.95-1.05 (m, 28H), 1.40 (s, 6H), 2.75 (t, *J* = 6.3 Hz), 3.13 (s, 3H), 3.16 (s, 3H), 3.23 (s, 3H), 3.60 (d, *J* = 11.4 Hz, 1H), 3.91-4.00 (m, 3H), 4.26-4.38 (m, 2H), 4.39-4.43 (m, 1H), 5.95 (s, 1H), 6.06 (d, *J* = 7.5 Hz, 1H), 8.39 (d, *J* = 7.5 Hz, 1H), 8.82 (s, 1H). ^{13}C NMR (100 MHz, $CDCl_3$): δ = 13.1, 13.3, 13.4, 13.5, 17.0, 17.1, 17.2, 17.3, 18.9, 23.8, 24.4, 35.1, 41.5, 48.8, 57.8, 64.6, 67.9, 81.2, 82.9, 88.6, 100.6, 101.9, 117.8,

141.6, 156.1, 158.5, 171.9. For the spectra, see page S17. Positive ion ESI-HRMS: *m/z* obsd. 706.3642 $[M+H]^+$; calcd. 706.3643 $[M+]^+$.

2'-*O*-(2-Cyanoethyl)-*N*4-(dimethylaminomethylene)-5'-*O*-(1-methoxy-1-methylethyl)-3'-*O*-(1,1,3,3-tetraisopropyldisiloxane-1-yl)adenosine (**3d**). Compound **2d** (284 mg, 0.447 mmol) was transformed to **3d** as described above for the transformation of **2a** to **3a.** Compound **3d** was obtained in 94% yield as white foam (300 mg; 0.424 mmol). ^{1}H NMR (400 MHz, $CDCl_3$): δ = 0.94-1.07 (m, 28H), 1.37 (s, 3H), 1.38 (s, 3H), 2.64 (br s, 2H), 3.16 (s, 3H), 3.18 (s, 3H), 3.23 (s, 3H), 3.60 (dd, *J* = 10.9 and 1.0 Hz, 1H), 3.77-3.92 (m, 3H), 4.26-4.28 (m, 1H), 4.47-4.49 (m, 1H), 4.72-4.73 (m, 1H), 6.18 (s, 1H), 8.31 (s, 1H), 8.48 (s, 1H), 8.88 (s, 1H). ^{13}C NMR (100 MHz, $CDCl_3$): δ = 13.2, 13.3, 13.4, 13.5, 17.1, 17.2, 17.2, 17.3, 18.9, 24.2, 24.4, 35.1, 41.3, 48.7, 59.4, 65.2, 70.4, 82.8, 83.0, 86.7, 100.6, 117.4, 126.5, 140.3, 150.9, 152.6, 158.1, 159.6. For the spectra, see page S18. Positive ion ESI-HRMS: *m/z* obsd. 708.3936 $[M+H]^+$; calcd. 708.3936 $[M+]^+$.

3-Benzoyl-2'-*O*-(2-cyanoethyl)-5'-*O*-(1-methoxy-1-methylethyl)uridine (**4a**). Compound **3a** (0.90 g, 1.22 mmol) was dissolved in dry MeOH (48 mL) and 3 equiv. of NH_4F (140 mg, 3.78 mmol) was added. The mixture was agitated for 30h at room temperature. The solution was then extracted with DCM (30 mL) and the organic phase was washed with aq. $NaHCO_3$ (100 mL). The aqueous phase was back-extracted twice with DCM (2 x 30mL) and the combined organic phase was dried over Na_2SO_4. Purification by column chromatography on silica gel using a gradient of 1-10% MeOH in DCM containing 1% pyridine afforded **4a** in 83% yield as white foam (0.48 g, 1.01 mmol). ^{1}H NMR (400 MHz, $CDCl_3$): δ = 1.40 (s, 3H), 1.41 (s, 3H), 1.52 (s, 3H), 2.62 (t, *J* = 6.1 Hz, 2H), 3.23 (s, 3H), 3.64-3.72 (m, 1H), 3.78-3.94 (3H), 3.95-4.15 (m, 3H), 4.19-4.24 (1H), 4.29-4.33 (m, 1H). 5.79 (d, *J* = 8.2 Hz, 1H), 5.86 (s, 1H), 7.50 (t, *J* = 7.4 Hz, 2H), 7.66 (t, *J* = 7.4 Hz, 1H), 7.93 (d, *J* = 7.4 Hz, 2H), 8.31 (d, *J* = 8.2 Hz, 1H). ^{13}C NMR (100 MHz, $CDCl_3$): δ = 18.9, 24.2, 24.3, 48.7, 57.9, 65.3, 67.8, 82.8, 83.0, 88.0, 100.4, 101.3, 117.7, 129.3, 130.5, 131.2, 135.4, 139.9, 149.3, 162.2, 168.7. For the spectra, see page S19. Positive ion ESI-HRMS: *m/z* obsd. 496.1692 $[M+Na]^+$; calcd. 496.1696 $[M+Na]^+$.

2'-*O*-(2-Cyanoethyl)-5'-*O*-(1-methoxy-1-methylethyl)uridine (**4a'**). Compound **3a** (4.69 g, 6.39 mmoles) was dissolved in dry MeOH (220 mL) and 5 equiv. of NH_4F (1.18 g, 31.9 mmol) was added. The mixture was agitated for 96 h at room temperature. The solvent was removed under reduced pressure and the residue was sujected to column chromatography on silica gel using a gradient of 1-5% MeOH in DCM containing 1% pyridine to afford **4a'** in 84% yield as white foam (1.98 g, 5.36 mmol). ^{1}H NMR (400 MHz, $CDCl_3$): δ = 1.40 (s, 6H), 2.73-2.77 (m, 2H), 3.22 (s, 3H), 3.70 (dd, *J* = 11.4 and 2.0 Hz, 1H), 3.88 (dd, *J* = 11.4 and 2.0 Hz, 1H), 3.92-3.97 (m, 2H), 4.10-4.14 (m, 1H), 4.18-4.24 (m, 1H), 4.27-4.31 (m, 1H), 5.69 (d, *J* = 8.2 Hz, 1H), 5.89 (s, 1H), 8.17 (d, *J* = 8.2 Hz, 1H), 10.2 (br s, 1H). ^{13}C NMR (100 MHz, $CDCl_3$): δ = 19.0, 24.2, 24.3, 48.7, 57.9, 65.3, 67.9, 82.8, 83.0, 87.7, 100.4, 101.7, 117.6,

139.8, 150.6, 163.7. For the spectra, see page S20. Positive ion ESI-HRMS: *m/z* obsd. 392.1429 [M+Na]$^+$; calcd. 392.1434 [M+Na].

2'-*O*-(2-Cyanoethyl)-*N*^{2}-(dimethylaminomethylene)-5'-*O*-(1-methoxy-1-methylethyl)guanosine (**4b**). Compound **3b** (1.52 g, 2.10 mmol) was dissolved in dry MeOH (83 mL), 5equiv. of NH_4F (390 mg, 10.52 mmol) was added and the mixture was agitated for 20 h at room temperature. Purification by column chromatography on silica gel using a gradient of 1-10% MeOH in DCM containing 1% triethylamine afforded **4b** in 85% yield as white foam (0.83 g, 1.79 mmol). ^{1}H NMR (400 MHz, $CDCl_3$): δ = 1.41 (s, 6H), 2.71 (t, *J* = 6.0 Hz, 2H), 3.09 (s, sH), 3.19 (s, 3H), 3.20 (s, 3H), 3.67 (dd, *J* = 11.0 and 3.3 Hz, 1H), 3.78-3.84 (m, 2H), 3.93-4.00 (m, 2H), 4.17-4.19 (m, 1H), 4.22-4.24 (m, 1H), 4.62-4.66 (m, 1H), 6.12 (s, 1H), 8.00 (s, 1H), 8.63 (s, 1H). ^{13}C NMR (100 MHz, $CDCl_3$): δ = 19.0, 24.3, 35.2, 41.5, 48.7, 59.7, 65.9, 69.9, 82.7, 83.6, 86.3, 100.4, 117.7, 120.1, 136.1, 149.9, 157.0, 158.1, 158.3. For the spectra, see page S21. Positive ion ESI-HRMS: *m/z* obsd. 464.2257 [M+H]$^+$; calcd. 464.2258 [M+]$^+$.

2'-*O*-(2-Cyanoethyl)-5'-*O*-(1-methoxy-1-methylethyl)cytidine (**4c′**). Compound **3c** (1.15 g, 1.68 mmol) was desilylated as described above for the desilylation of **3b**. Purification by column chromatography on silica gel using a stepwise gradient of 1-14% MeOH in DCM containing 1% triethylamine gave **4c′** in 80 % yield (0.50 g, white foam). ^{1}H NMR (400 MHz, $CDCl_3$): δ = 1.40 (s, 3H), 1.41 (s, 3H), 2.75 (t, *J* = 6.1 Hz, 2H), 3.22 (s, 3H), 3.69 (dd, *J* = 11.3 and 2.0 Hz, 1H), 3.88 (dd, *J* = 11.3 and 2.0 Hz, 1H), 3.94-4.02 (m, 2H), 4.09-4.13 (m, 1H), 4.21-4.30 (m, 2H), 5.75 (d, *J* = 7.5 Hz, 1H), 5.88 (s, 1H), 8.19 (d, *J* = 7.5 Hz, 1H). ^{13}C NMR (100 MHz, $CDCl_3$): δ = 19.0, 24.3, 48.7, 58.0, 65.1, 67.7, 82.4, 82.8, 88.4, 93.2, 100.3, 117.8, 141.2, 155.7, 165.8. For the spectra, see page S22. Negative ion ESI-HRMS: *m/z* obsd. 367.1597 [M-H]$^-$, 403.1389 [M+Cl]$^-$; calcd. 367.1623 [M-H]$^-$, 403.1384 [M+Cl]$^-$.

***N*^{4}-Benzoyl-2'-*O*-(2-cyanoethyl)-5'-*O*-(1-methoxy-1-methylethyl)cytidine** (**4c″**). Compound **4c′** (0.35 g, 0.95 mmol) was dissolved in dry pyridine (45 mL) and 1.1 equiv. of benzoyl chloride (121 µL, 1.04 mmol) was added. The mixture was agitated for 20 h at room temperatura and subjected then to DCM/aq. $NaHCO_3$ workup. Purification by column chromatography on silica gel using a stepwise gradient of 1-6% MeOH in DCM containing 1% triethylamine gave **4c″** in 91% yield (0.41 g, white foam). ^{1}H NMR (400 MHz, $CDCl_3$): δ = 1.43 (s, 3H), 1.44 (s, 3H), 2.77 (t, *J* = 5.6 Hz, 2H), 3.24 (s, 3H), 3.73 (d, *J* = 10.9 Hz, 1H), 3.93 (d, *J* = 10.9 Hz, 1H), 3.98-4.06 (m, 2H), 4.16-4.19 (m, 1H), 4.27-4.30 (m, 1H), 4.33-4.37 (m, 1H), 5.93 (s, 1H), 7.50-7.57 (m, 3H), 7.62 (t, *J* = 7.5 Hz, 1H), 7.92 (d, *J* = 7.5 Hz, 2H), 8.42 (d, *J* = 7.4 Hz, 1H), 9.05 (br s, 1H). ^{13}C NMR (100 MHz, $CDCl_3$): δ = 19.0, 24.3, 24.5, 48.7, 57.5, 65.2, 67.3, 82.6, 82.8, 88.8, 96.0, 100.4, 117.7, 127.6, 129.0, 132.9, 133.2, 144.9, 154.9, 162.7, 166.6. For the spectra, see page S23. For Negative ion ESI-HRMS: *m/z* obsd. 471.1862 [M-H]$^-$, 507.1629 [M+Cl]$^-$; calcd. 471.1881 [M-H]$^-$, 507.1647 [M+Cl]$^-$.

2'-*O*-(2-Cyanoethyl)-*N*^{4}-(dimethylaminomethylene)-5'-*O*-(1-methoxy-1-methylethyl)adenosine (**4d**). Compound **3d** (1.20 g, 1.69 mmol) was desilylated as described above for the desilylation of **3b** to **4b**. Purification by column chromatography on silica gel using a gradient of 1-5% MeOH in DCM containing 1% triethylamine afforded **4d** as white foam in 63% yield (0.48 g; 1.07 mmol). In addition, 2'-*O*-(2-cyanoethyl)-5'-*O*-(1-methoxy-1-methylethyl)adenosine (0.14g, 0.35 mmol) was obtained. Treatment of this compound in MeOH (10 mL) with *N,N*-dimethylformamide dimethyl acetal (151 µL, 0.86 mmol) for 72 h at room temperature yielded additional 0.33 mmol of **4d**. Accordingly,**4b** was obtained in 83% overall yield. ^{1}H NMR (400 MHz, $CDCl_3$): δ = 1.42 (s, 3H), 1.43 (s, 3H), 2.75 (m, *J* = 5.6 Hz, 2H), 3.21 (s, 6H), 3.26 (s, 3H), 3.70 (d, *J* = 10.6 Hz, 1H), 3.87 (d, *J* = 10.6 Hz, 1H), 3.86-3.95 (m, 1H), 4.10-4.14 (m, 1H), 4.22-4.24 (m, 1H), 4.29-4.30 (m, 1H), 4.52-4.53 (m, 1H), 6.24 (s, 1H), 8.39 (s, 1H), 8.51 (s, 1H), 8.95 (s,1H). ^{13}C NMR (100 MHz, $CDCl_3$): δ = 19.0, 24.3, 35.2, 41.3, 48.7, 59.1, 65.6, 69.3, 83.0, 83.1, 86.8, 100.4, 117.3, 126.4, 139.7, 150.8, 152.6, 158.1, 159.6. For the spectra, see page S24. Positive ion ESI-HRMS: *m/z* obsd. 448.2327 $[M+H]^+$; calcd. 448.2308 $[M+H]^+$.

2'-*O*-(2-Cyanoethyl)-5'-*O*-(1-methoxy-1-methylethyl)uridine 3'-(2-cyanoethyl-*N*,*N*-diisopropyl)phosphoramidite (**5a´**). Compound **4a'** (1.40 g, 3.79 mmol) was dissolved in dry DCM (15 mL) under N_2. *N,N*-Diisopropylethylamine (0.95 mL, 5.45 mmol) and 1-chloro-1-(2-cyanoethoxy)-*N*,*N*-diisopropylphosphanamine (0.93 mL, 4.16 mmol) were added and the mixture was stirred for 3 h at room temperarure. The solution was passed through a short silica gel column, which was first eluted with DCM containing 1% triethylamine and then with a 84:15:1 mixture *(v/v/v)* EtOAc, petroleum ether and triethylamine. **5a´** was obtained in 95% yield as white foam (2.05 g, 3.60 mmol). ^{1}H-NMR (400 MHz, $CDCl_3$): δ = 1.17-1.28 (m, 14H), 1.39 (s, 6H), 2.62-2.75 (m, 4H), 3.22 (s, 3H), 3.52-4.35 (m, 9H), 5.66 (d, *J* = 8.1 Hz, 1H), 5.88 (s, 0.6H), 5.90 (s, 0.4H), 8.16 (m, 1H). ^{13}C NMR (100 MHz, $CDCl_3$): δ = 19.0, 24.1, 24.6, 48.9, 58.1, 65.5, 69.3, 81.4, 82.5, 87.7, 100.4, 101.7, 117.9, 139.8, 150.6, 163.6. ^{31}P NMR ($CDCl_3$) : 149.0 (40%), 150.2 (60%). For the ^{31}P NMR spectrum, see page S25. Positive ion ESI-HRMS: *m/z* obsd. 592.2511 $[M+Na]^+$; calcd. 592.2512 $[M+Na]^+$.

2'-*O*-(2-Cyanoethyl)-*N*^{2}-(dimethylaminomethylene)-5'-*O*-(1-methoxy-1-methylethyl)guanosine 3'-(2-cyanoethyl-*N,N*-diisopropyl)phosphoramidite (**5b**). Compound **4b** (0.49 g, 1.05 mmol) was phosphitylated to **5b** as described above for the phosphtylation of **4a** to **5a**. The product was purified by passing the mixture through a short silica gel using acetone that contained 1% *N,N*-diisopropylethylamine a eluent. **5b** was obtained as white foam in 88% yield (0.62 g; 0.92 mmol). ^{1}H NMR (400 MHz, CDCl3): δ = 1.20-1.27 (m, 14H), 1.42 (s, 6H), 2.60-2.67 (m, 4H), 3.10 (s, 3H), 3.18 (s, 3H), 3.20 (s, 3H), 3.57-4.40 (m, 8H), 4.53-4.58 (m, 1H), 6.12 (s, 1H),8.05 (s, 0.4H), 8.07 (s, 0.6H), 8.65 (s, 0.4H), 8.87 (s, 0.6H), 8.85 (br s, 1H); ^{13}C NMR (100 MHz, $CDCl_3$): δ = 19.0, 24.5, 35.1, 41.3, 48.8, 58.1, 66.0, 69.5, 81.9, 83.2, 86.9, 100.6, 117.8, 120.6, 135.8, 149.6, 156.9, 157.6, 158.3. ^{31}P NMR ($CDCl_3$) :

149.5 (40%), 150.7 (60%). For the ^{31}P NMR spectrum, see page S25. Positive ion ESI-HRMS: *m/z* obsd. 664.3337 [M+H]$^{+}$; calcd. 664.3336 [M+H]$^{+}$.

***N*^{4}-Benzoyl-2'-*O*-(2-cyanoethyl)-5'-*O*-(1-methoxy-1-methylethyl)cytidine 3'-(2-cyanoethyl-*N*,*N*-diisopropyl)phosphoramidite** (**5c´´**). Compound **4c´´** (0.41 g, 0.87 mmol) was phosphitylated to **5c** as described above for the phosphitylation of **4a** to **5a**. The crude mixture was passed through a short silica gel column by using acetone that contained 1% *N*,*N*-diisopropylethylamine as eluent. **5c´´** was obtained as white foam in 91% yield (0.53 g; 0.79 mmol). ^{1}H NMR (400 MHz, $CDCl_3$): δ = 1.14-1.30 (m, 14H), 1.43 (s, 3H), 1.46 (s, 3H), 2.60-2.74 (m, 4H), 3.25 (s, 3H), 3.72-4.35 (m, 9H), 5.94 (s, 1H), 7.49-7.54 (3H, m), 7.61 (m, 1H), 7.91 (m, 2H), 8.72 (m, 1H), 8.81 (br s, 1H); ^{13}C NMR (100 MHz, $CDCl_3$): δ = 19.0, 24.3, 48.9, 57.5, 65.3, 69.5, 81.8, 82.2, 89.8, 95.9, 100.4, 117.4, 117.9, 127.5, 129.1, 133.0, 133.2, 144.9, 154.8, 162.4, 166.6. ^{31}P NMR ($CDCl_3$) 149.0 (40%), 150.0 (60%). For the ^{31}P NMR spectrum, see page S26. Negative ion ESI-HRMS: *m/z* obsd. 671.2964 [M-H]$^{-}$; calcd. 671.2958 [M-H]$^{-}$.

2'-*O*-(2-Cyanoethyl)-*N*^{4}-(dimethylaminomethylene)-5'-*O*-(1-methoxy-1-methylethyl)adenosine 3'-(2-cyanoethyl-*N*,*N*-diisopropyl)phosphoramidite (**5d**). Compound **4d** (0.22 g, 0.49 mmol) was phosphitylated to **5d** as described above for the phosphitylation of **4a** to **5**, dissolved in dry DCM (10 mL). The crude product mixture was passed through a short silica gel column eluting first with EtOAc containing 1% *N*,*N*-diisopropylethylamine and then with with acetone also containing as 1% *N*,*N*-diisopropylethylamine. Later a constant gradient using 1% N,N-diisopropylethylamine. **5d** was obtained as white foam in 92% yield (0.293 g; 0.45 mmol). ^{1}H NMR (400 MHz, $CDCl_3$): δ = 1.19-1.26 (m, 14H), 1.39 (s, 3H), 1.41 (s, 3H), 2.62-2.70 (m, 4H), 3.19 (s, 3H), 3.21 (s, 3H), 3.26 (s, 3H), 3.60-4.00 (m, 6H), 4.36-4.64 (m, 3H), 6.21 (br s, 1H), 8.35 (s, 0.5H), 8.37 (s, 0.5H), 8.53 (s, 1H), 8.94 (s, 1H). ^{13}C NMR (100 MHz, CDCl3): δ = 19.1, 24.5, 35.2, 41.3, 48.7, 59.1, 65.6, 69.5, 82.3, 82.6, 87.5, 100.5, 117.4, 126.6, 140.1, 151.1, 152.7, 157.9, 159.7. ^{31}P NMR ($CDCl_3$) : 149.6 (45%), 149.9 (55%). For the ^{31}P NMR spectrum, see page S26. Positive ion ESI-HRMS: *m/z* obsd. 648.3388 [M+H]$^{+}$; calcd. 648.3387 [M+H]$^{+}$.

***N*^{3}-Benzoyl-2'-*O*-(2-cyanoethyl)-3'-*O*-(pent-4-ynoyl)-5'-*O*-(1-methoxy-1-methylethyl)uridine** (**6a**). 4-Pentynoic acid (0.32 g, 3.56 mmol) was dissolved in dry dioxane (5 mL) and the solution obtained was added dropwise to a solution of DCC (0.36 g, 1.78 mmol) in dioxane (10 mL) on an ice-bath. The mixture was stirred for 2 h at room temperature, filtered and concentrated by evaporation. 4-Pentynoic anhydride obtained was then added to a solution of **4a** (0.49 g, 1.03 mmol) in pyridine (20 mL) on an ice-bath. A catalytic amount of DMAP was added and the mixture was stirred for 2 h at room temperature. After completion, the solvent was removed by evaporation and the yellowish oil was subjected to chromatographic purification on a silica gel column using a gradient of 1-2% MeOH in DCM containing 1% TEA as an eluent. **6a** was obtained in 95% yield (0.544 g, 0.983 mmol). ^{1}H NMR (500 MHz, $CDCl_3$): δ = 1.19 (s, 3H), 1.22 (s, 3H), 2.29-2.53 (m, 4H), 2.77 (m, 2H), 2.90 (s, 1H), 3.00 (s,

3H), 3.44 (dd, *J* = 11.4 and 1.4 Hz, 1H), 3.51-3.55 (m, 1H), 3.70 (dd, *J* = 11.4 and 1.9 Hz, 1H), 3.74-3.78 (m, 1H), 4.10-4.14 (m, 1H), 4.26 (d, *J* = 8.0 Hz, 1H), 4.88-4.91 (m, 1H), 5.60 (d, *J* = 8.2 Hz, 1H), 5.72 (s, 1H), 7.31 (t, *J* = 7.4 Hz, 2H), 7.46 (t, *J* = 7.4 Hz, 1H), 7.75 (d, *J* = 7.4 Hz, 2H), 8.04 (d, *J* = 8.2 Hz, 1H), 8.65 (br s, 1H); ^{13}C NMR (125 MHz, $CDCl_3$): δ = 14.2, 18.8, 24.3, 32.8, 48.8, 58.0, 65.5, 69.3, 69.7, 80.7, 81.0, 82.2, 88.6, 100.6, 101.8, 117.5, 128.3, 129.3, 130.5, 135.3, 139.4, 149.3, 161.9, 168.5, 171.3. For the spectra, see page S27. Positive ion ESI-HRMS: *m/z* obsd. 576.1965 $[M+Na]^+$; calcd. 576.1958 $[M+Na]^+$.

^{1}H NMR. Compound **2a**

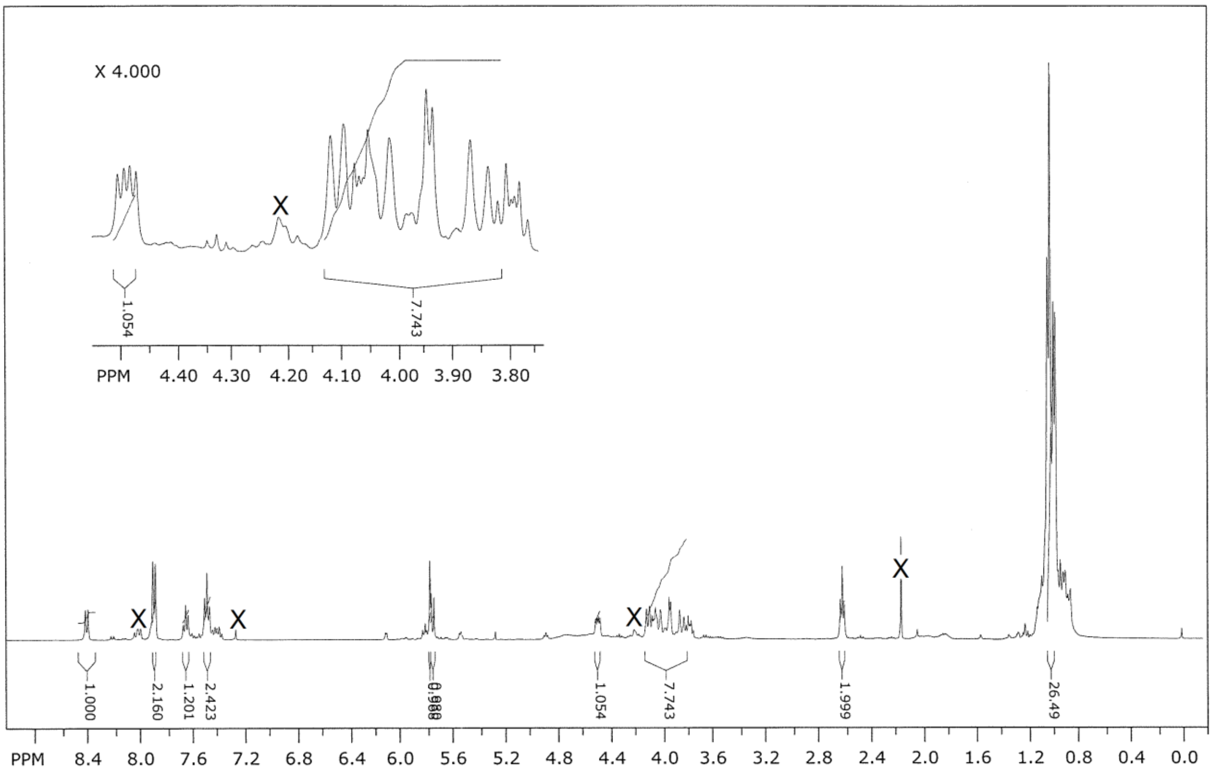

^{13}C NMR. Compound **2a**

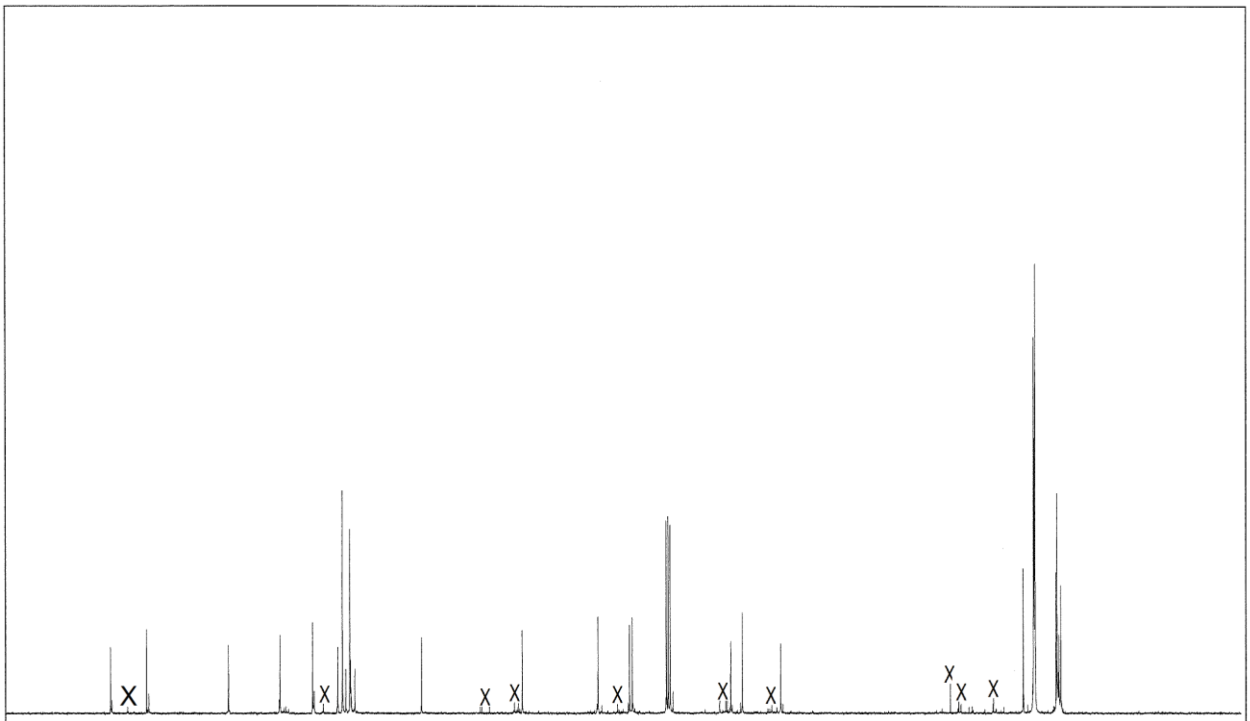

^{1}H NMR. Compound **2b**

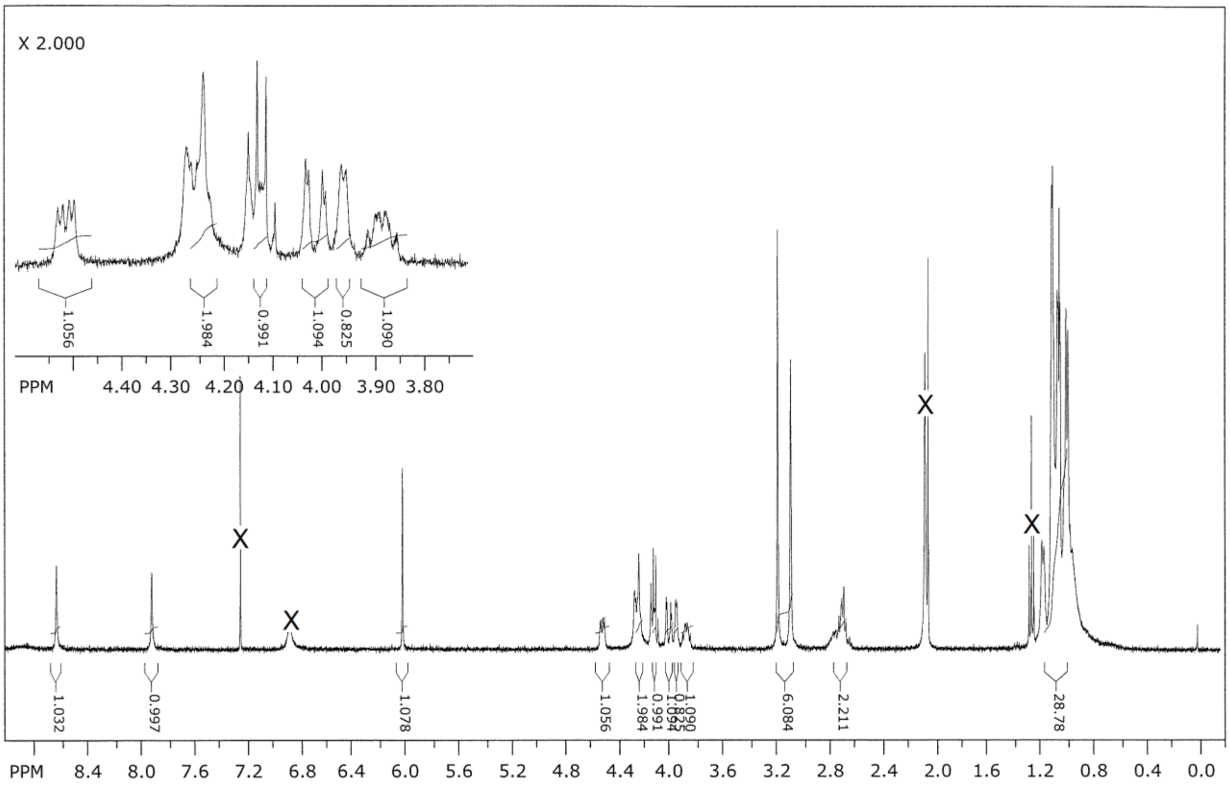

^{13}C NMR. Compound **2b**

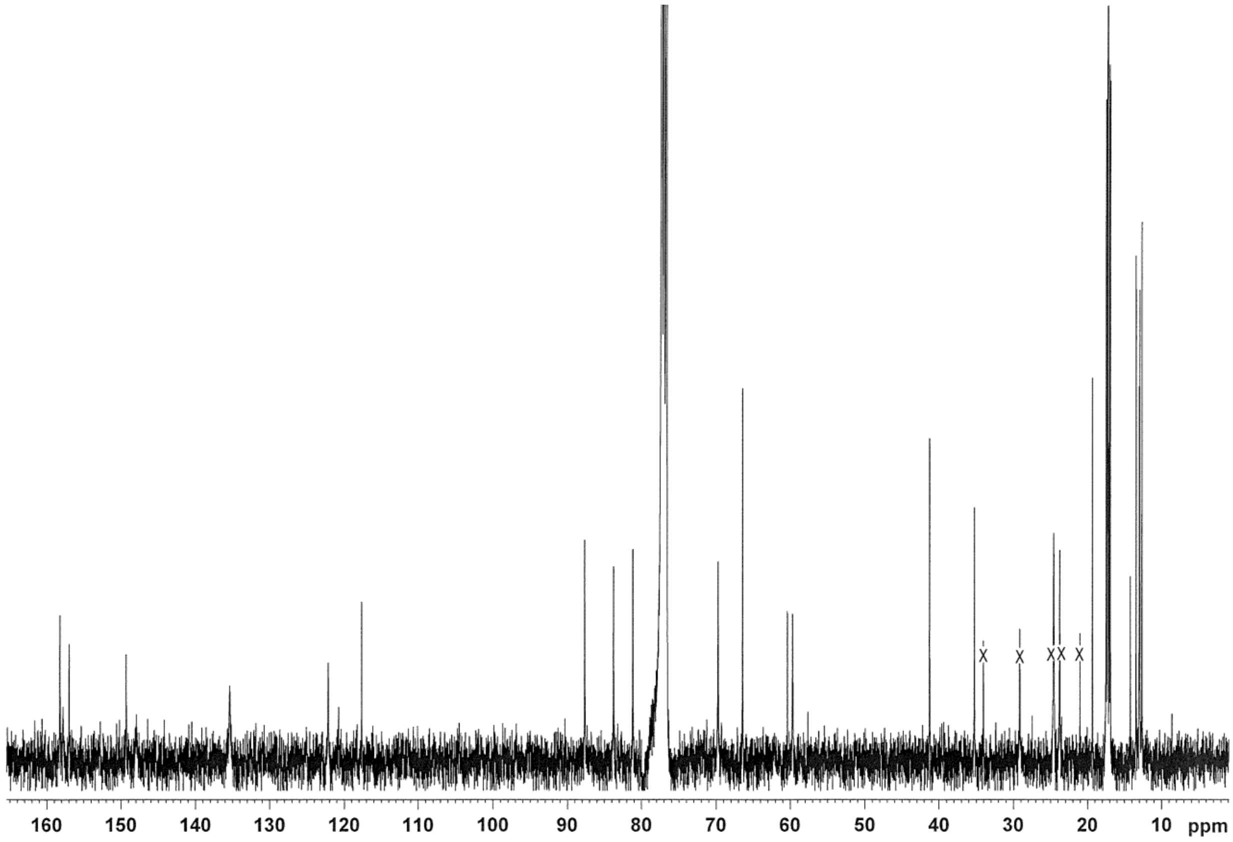

^{1}H NMR. Compound **2c**

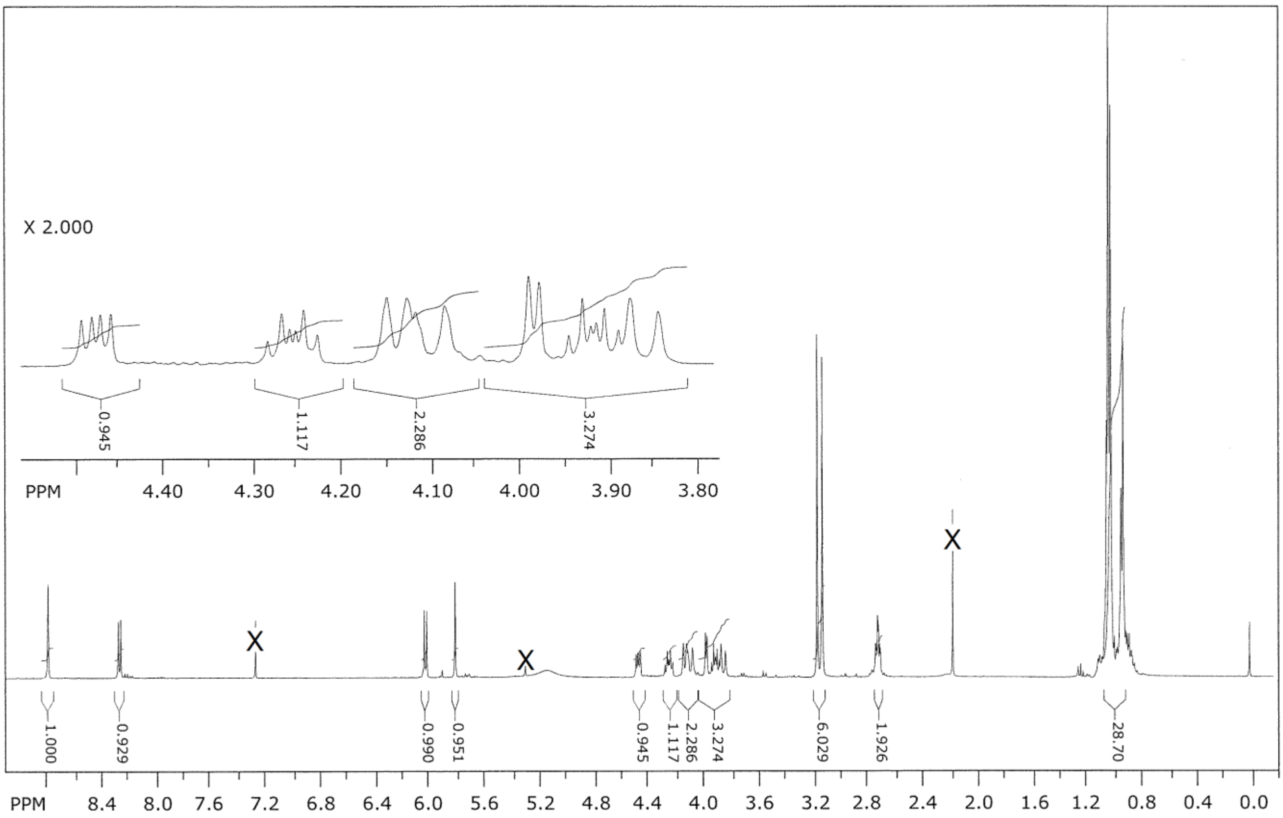

^{13}C NMR. Compound 2c

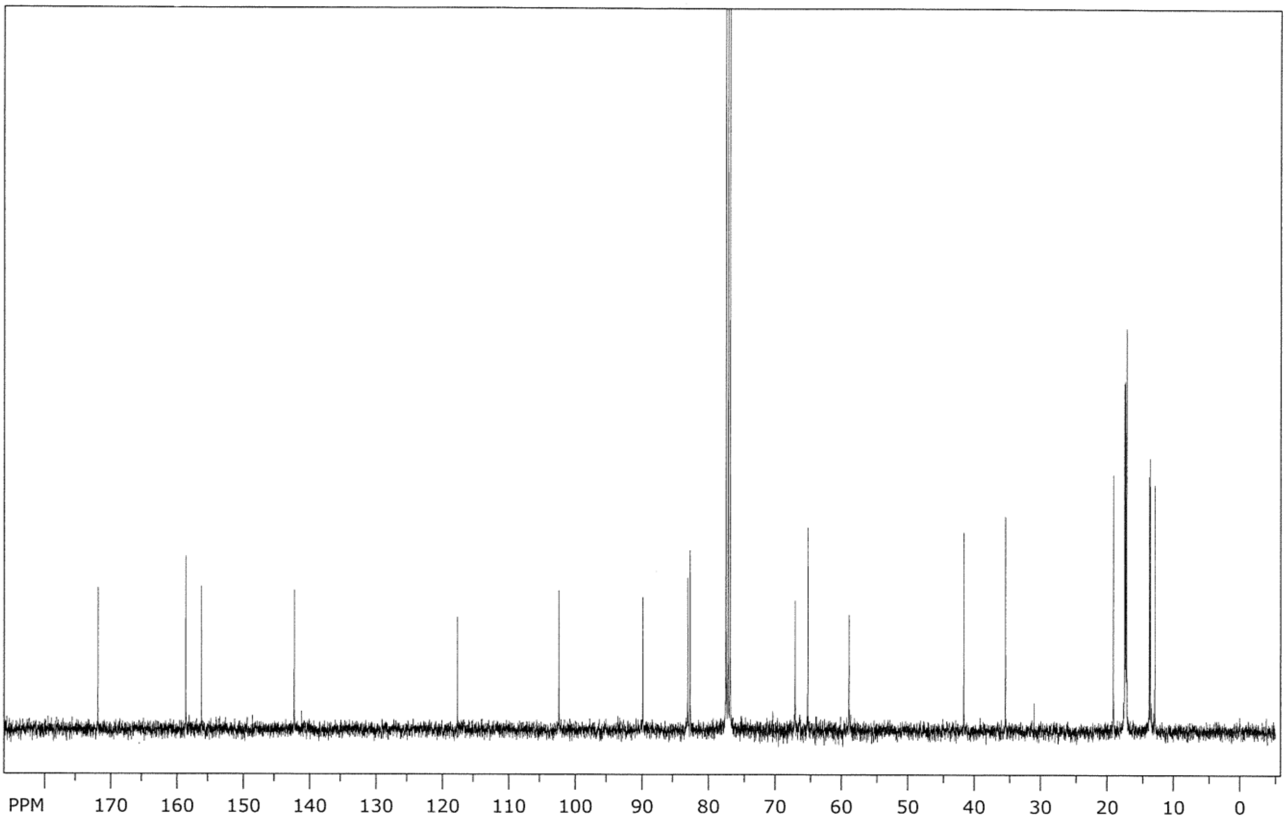

^{1}H NMR. Compound **2d**

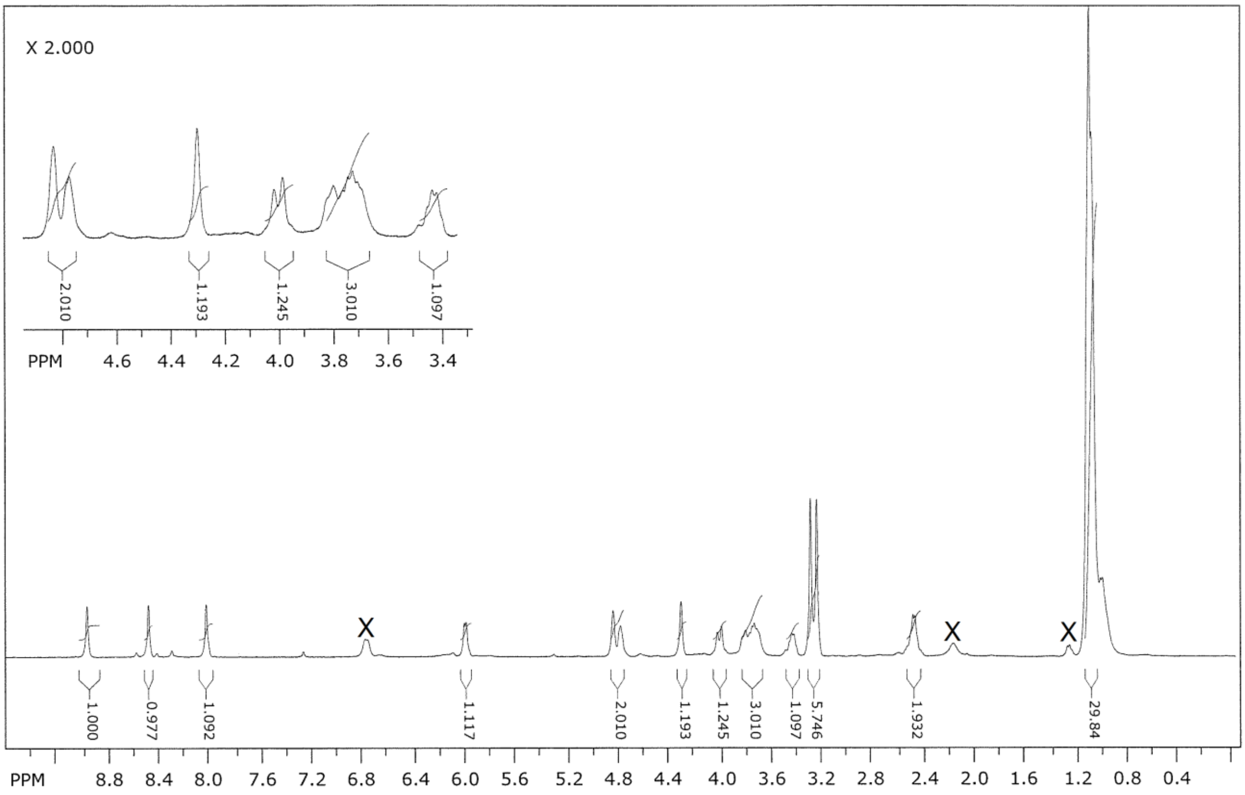

^{13}C NMR. Compound **2d**

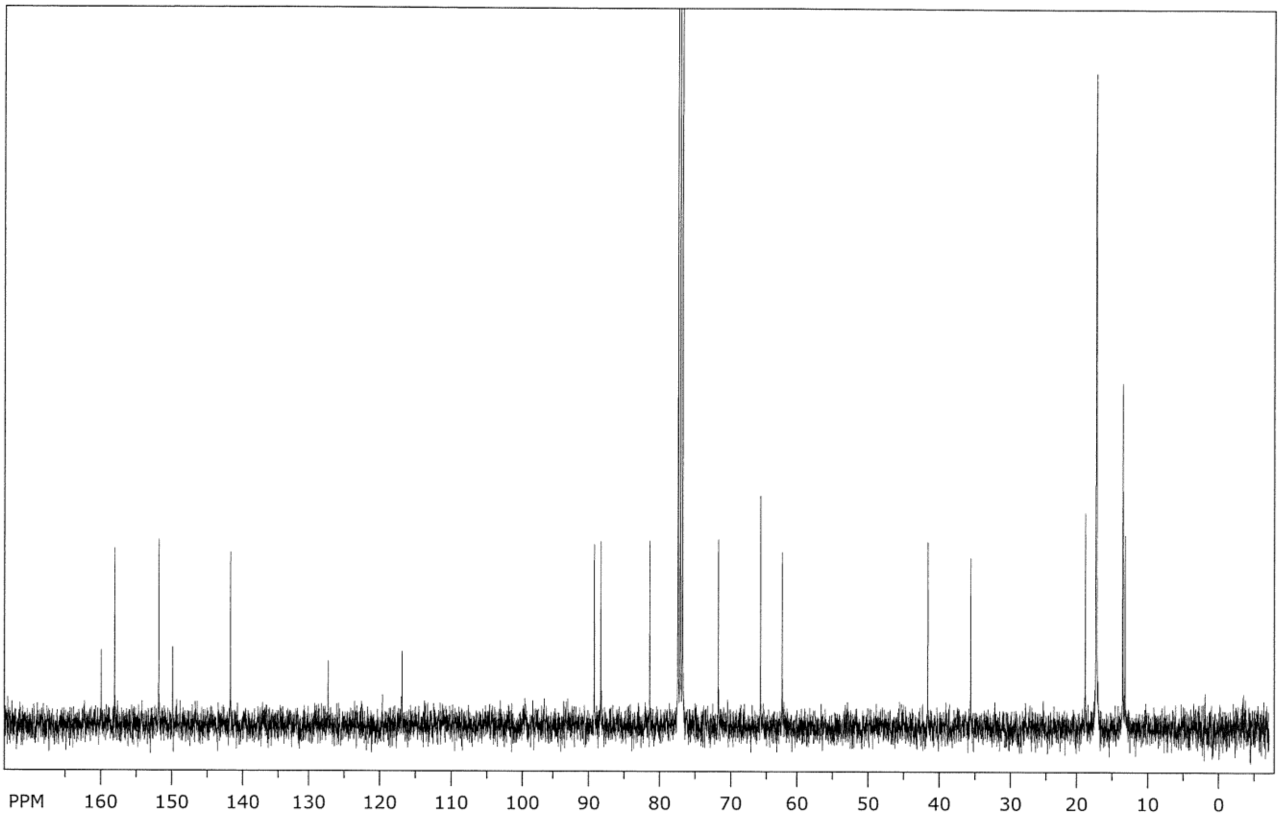

^{1}H NMR. Compound **3a**

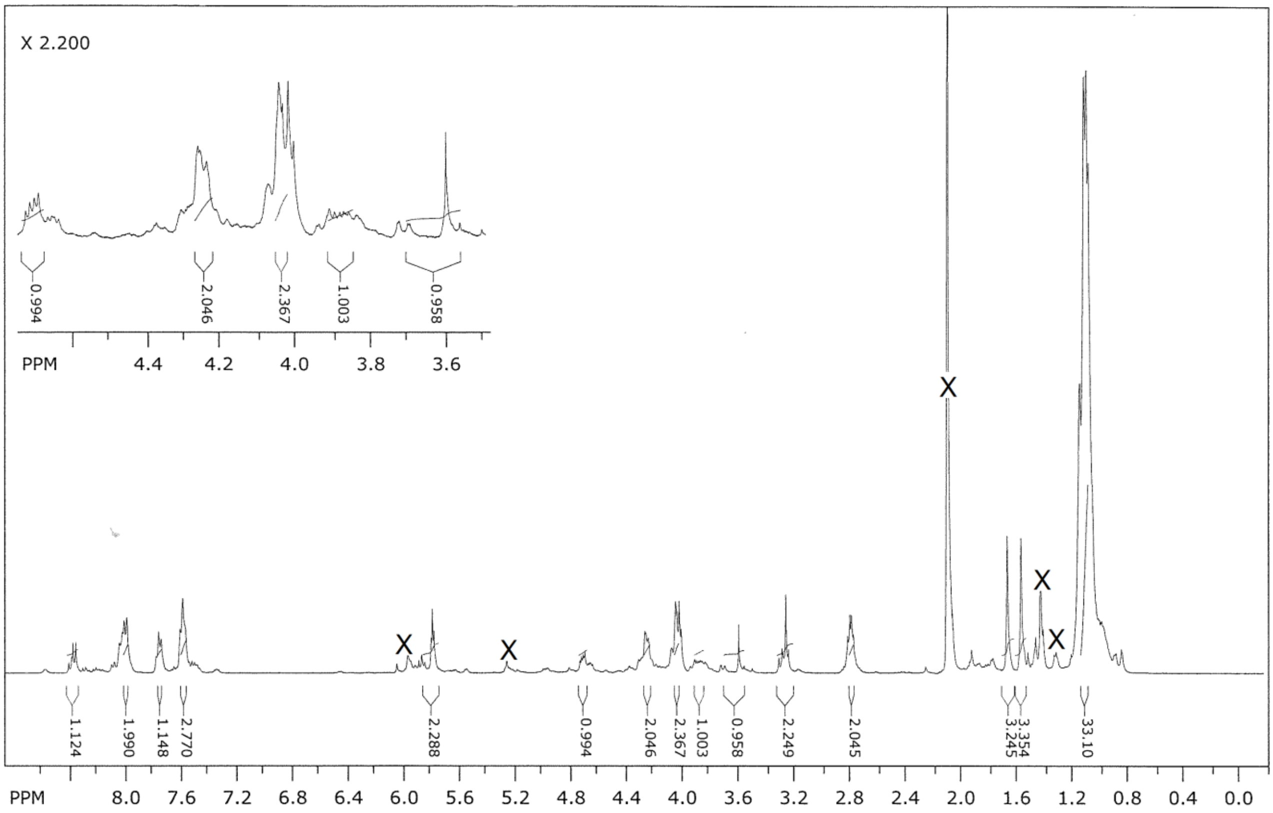

^{13}C NMR. Compound **3a**

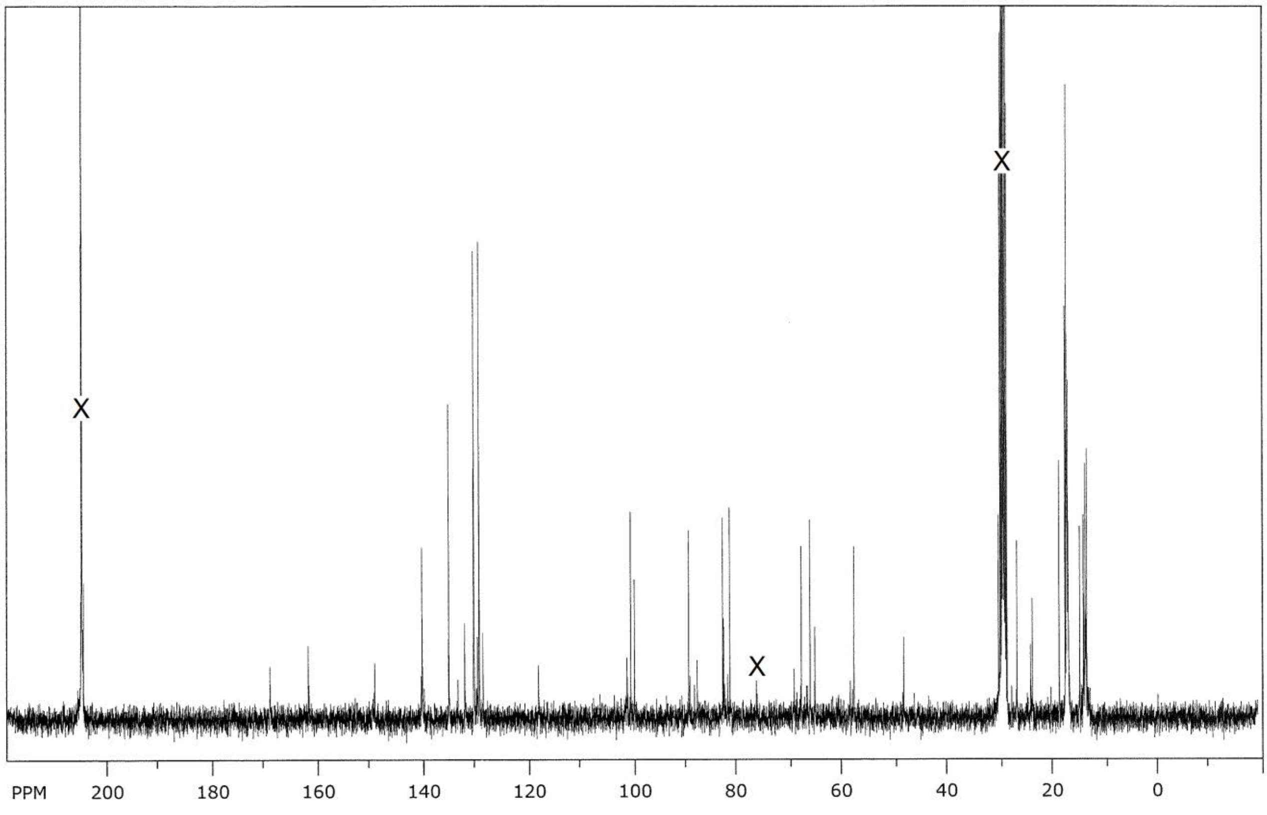

^{1}H NMR. Compound **3b**

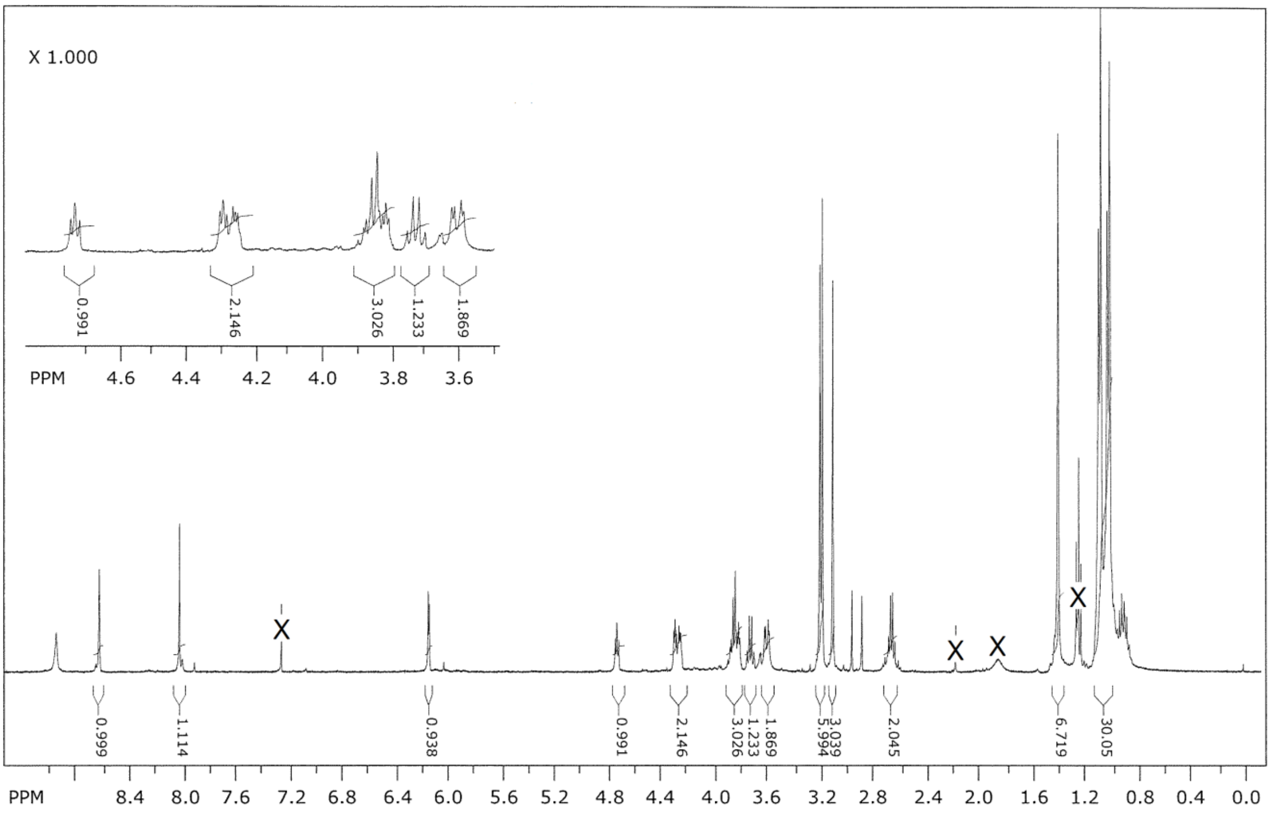

^{13}C NMR. Compound **3b**

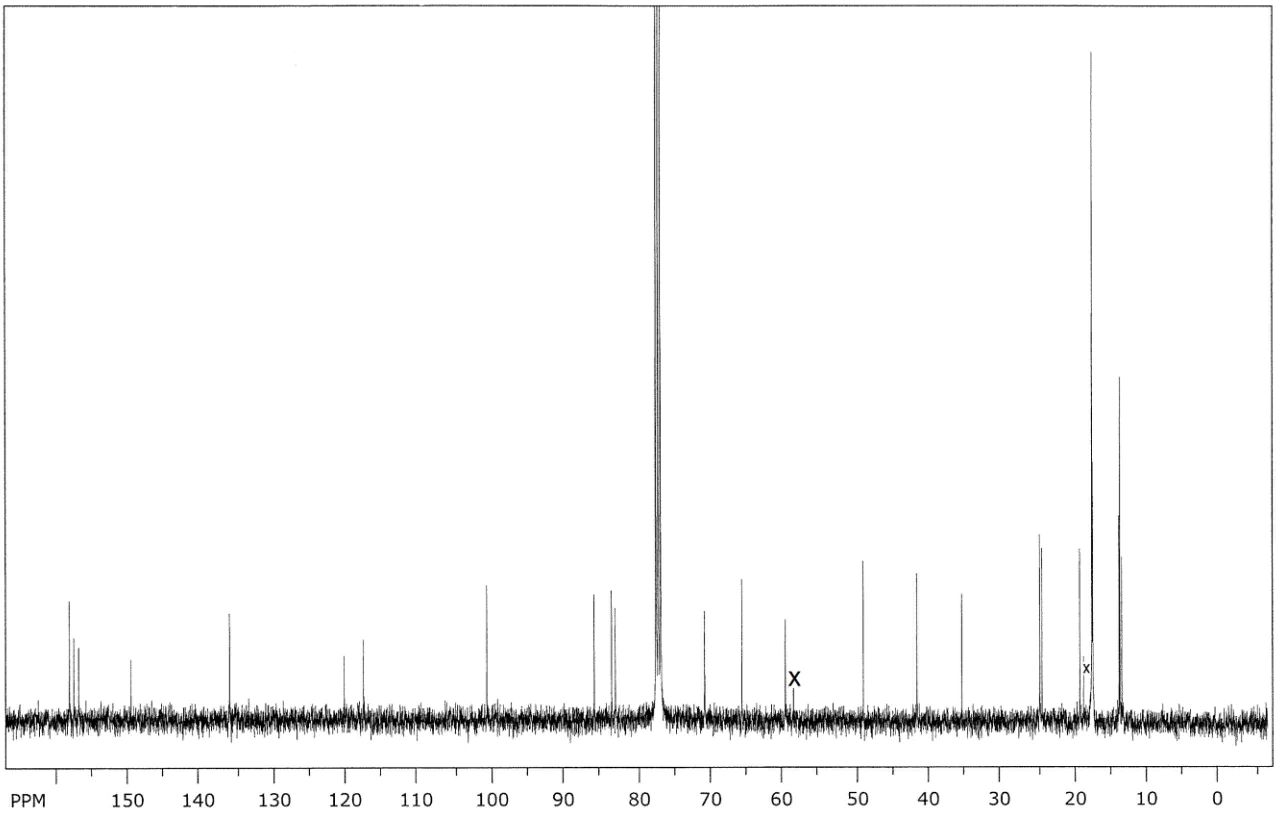

^{1}H NMR. Compound **3c**

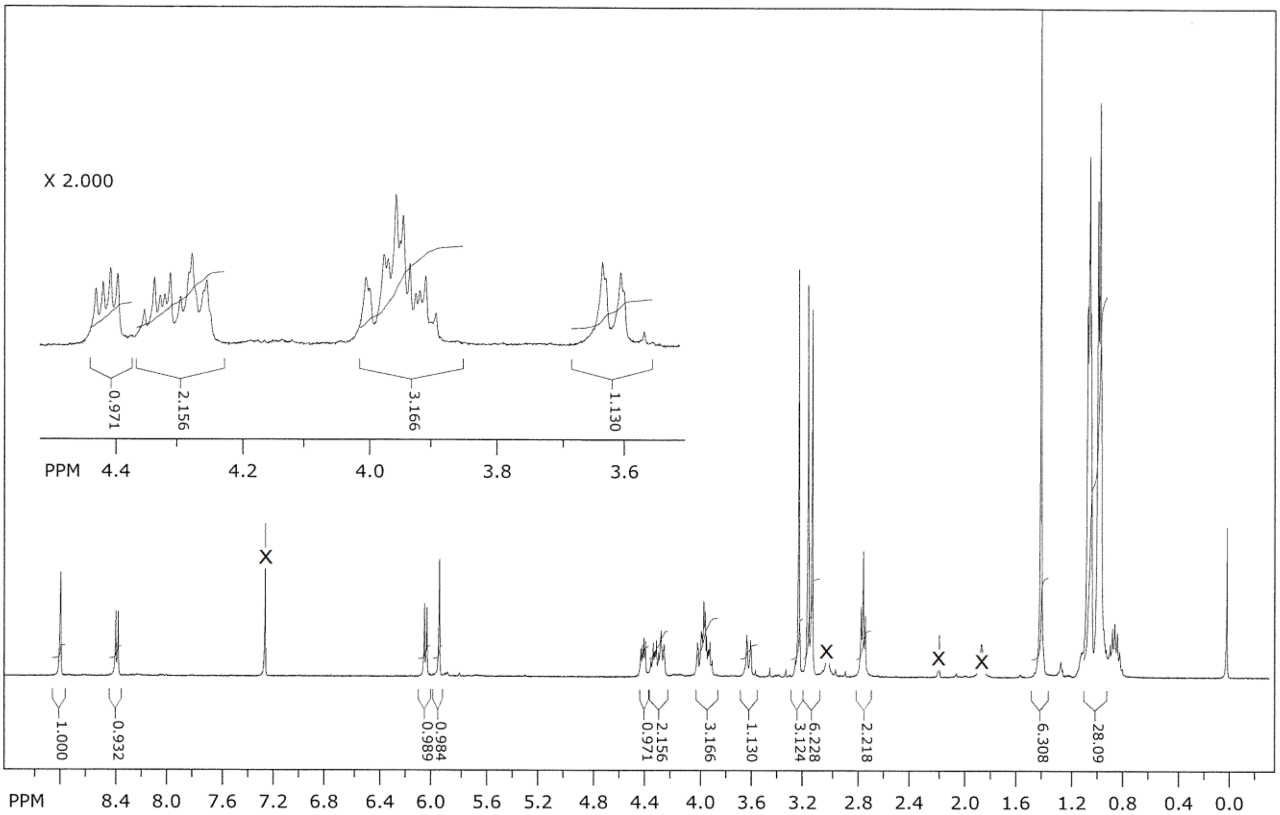

^{13}C NMR. Compound **3c**

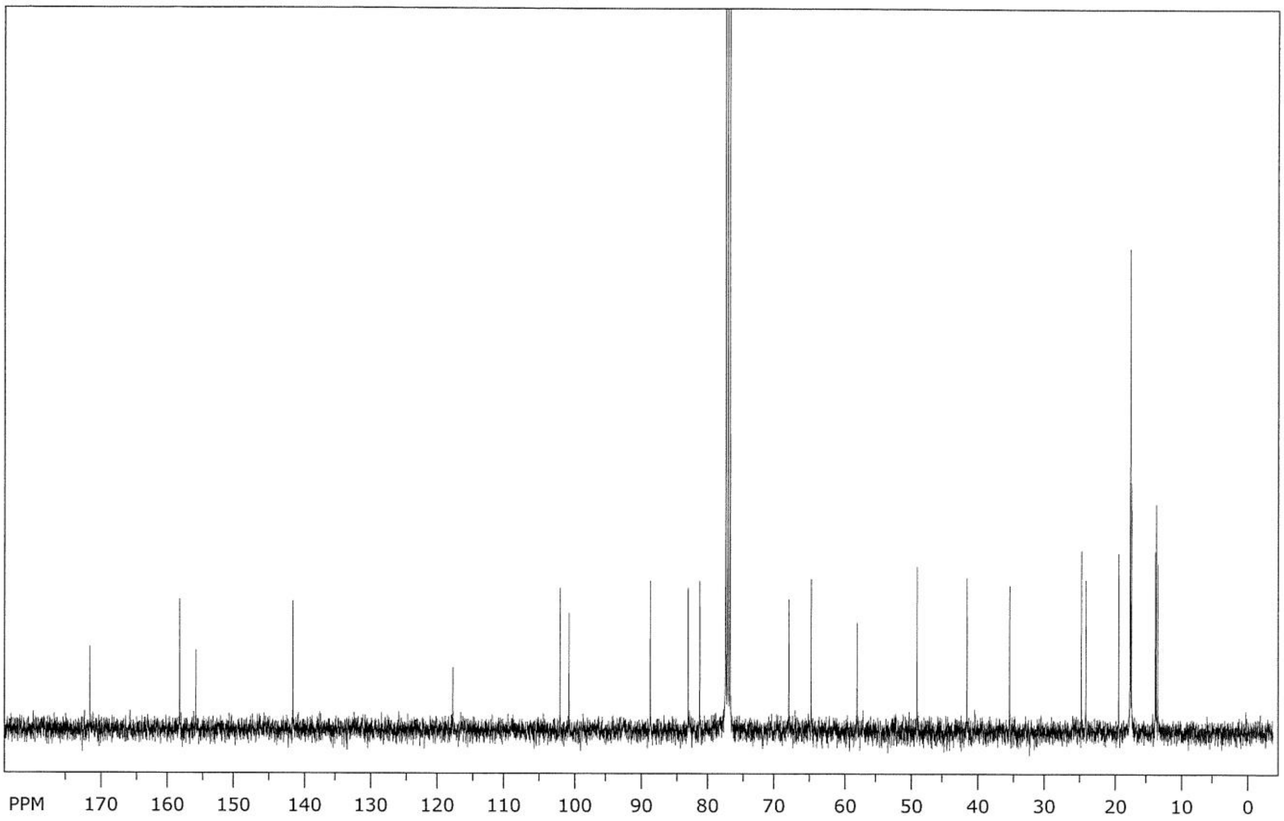

^{1}H NMR. Compound **3d**

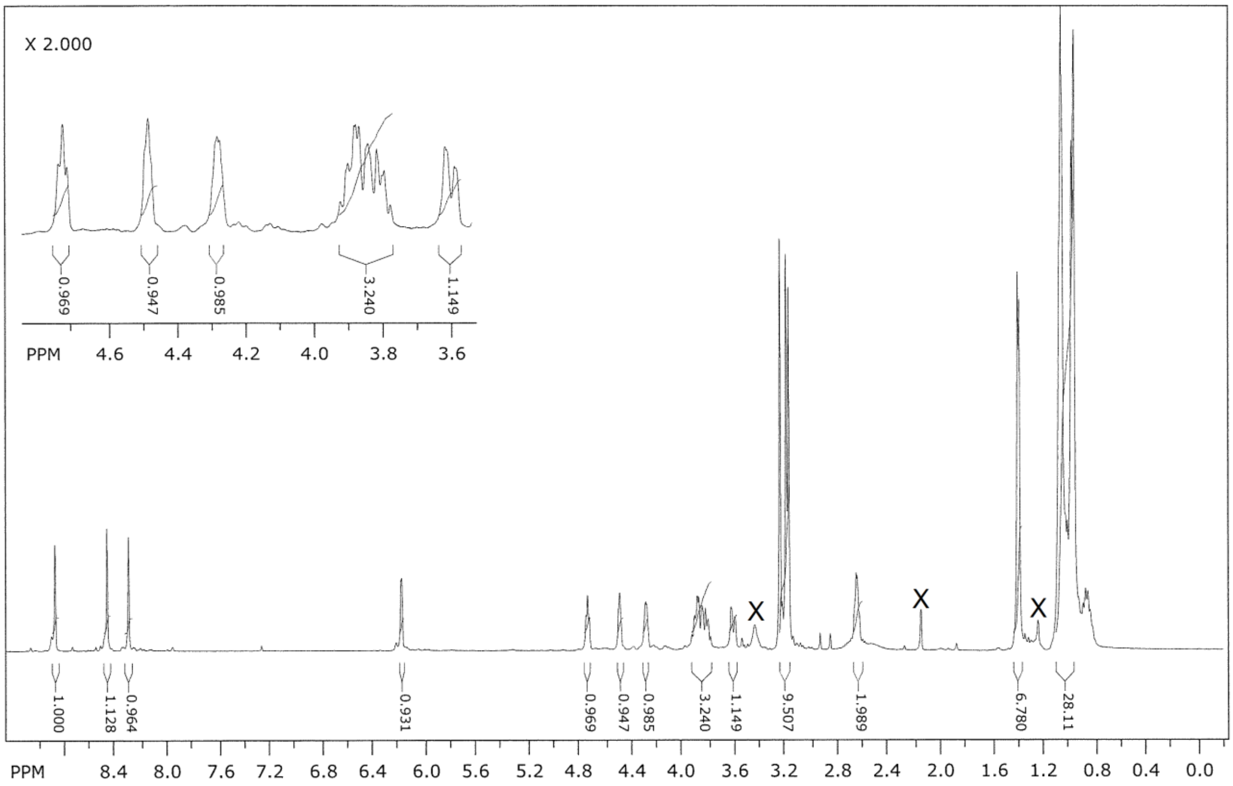

^{13}C NMR. Compound **3d**

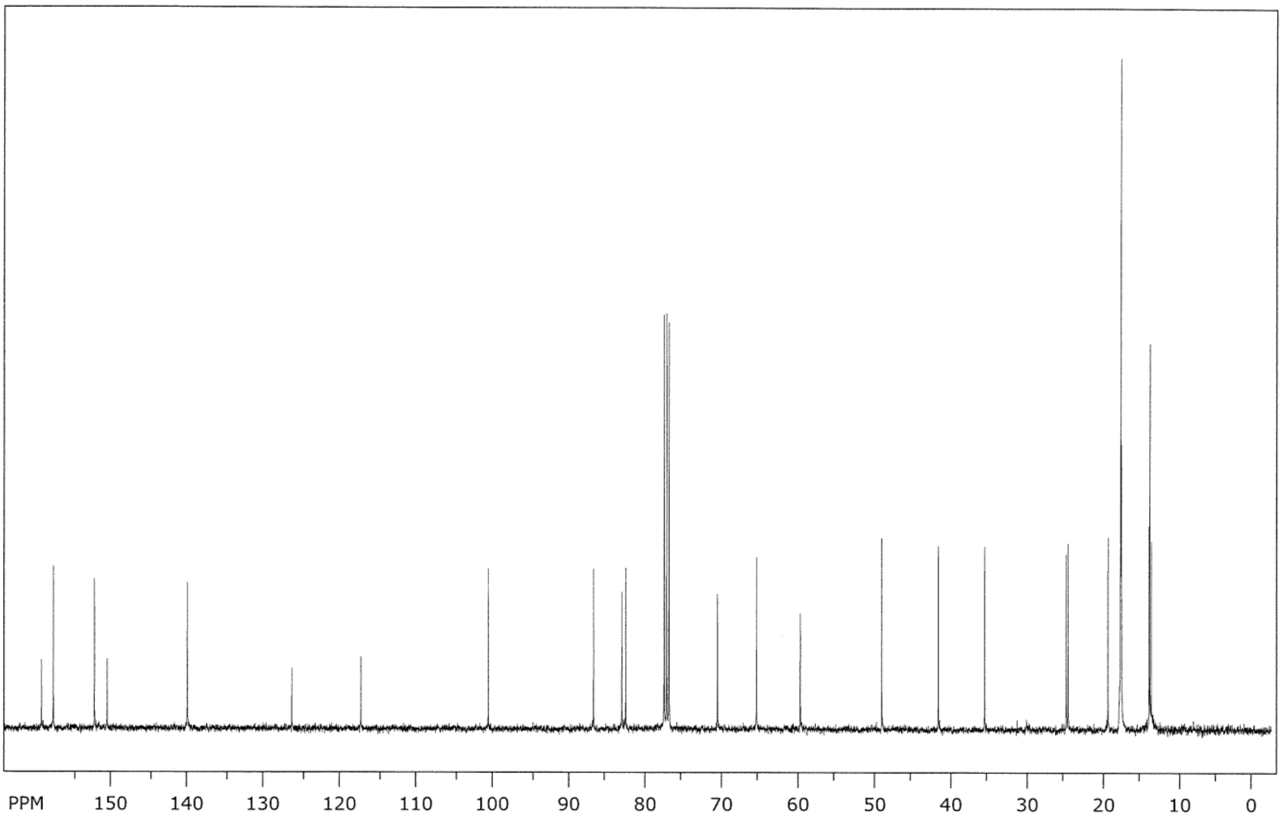

^{1}H NMR. Compound **4a**

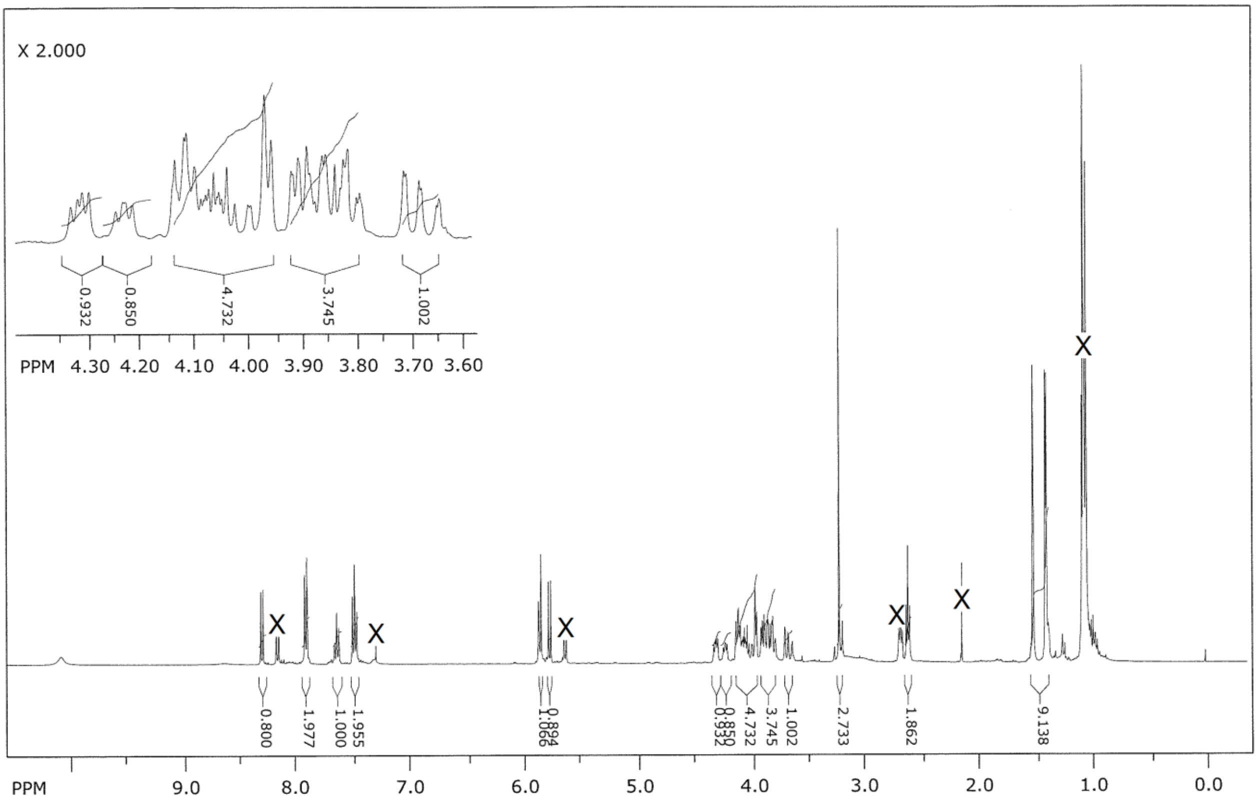

^{13}C NMR. Compound **4a**

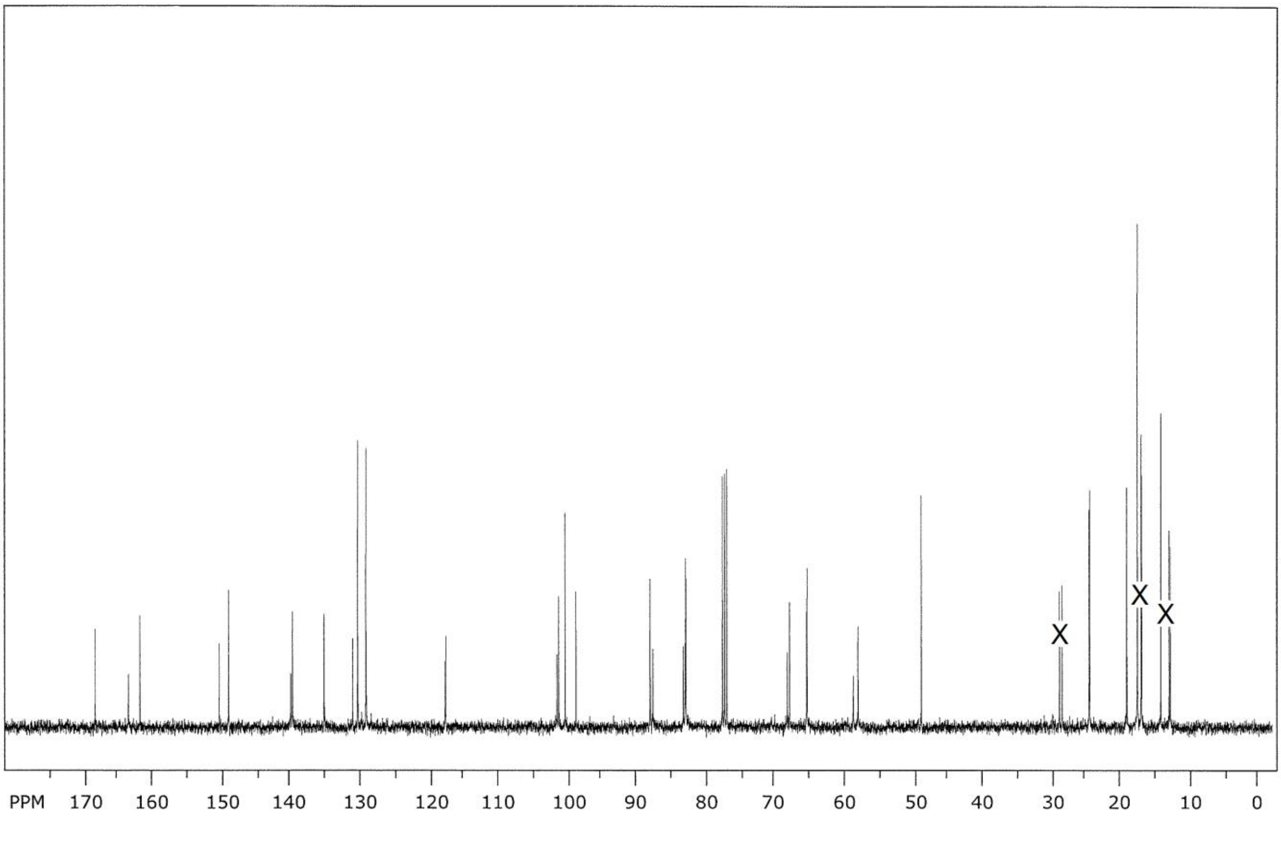

^{1}H NMR. Compound **4a’**

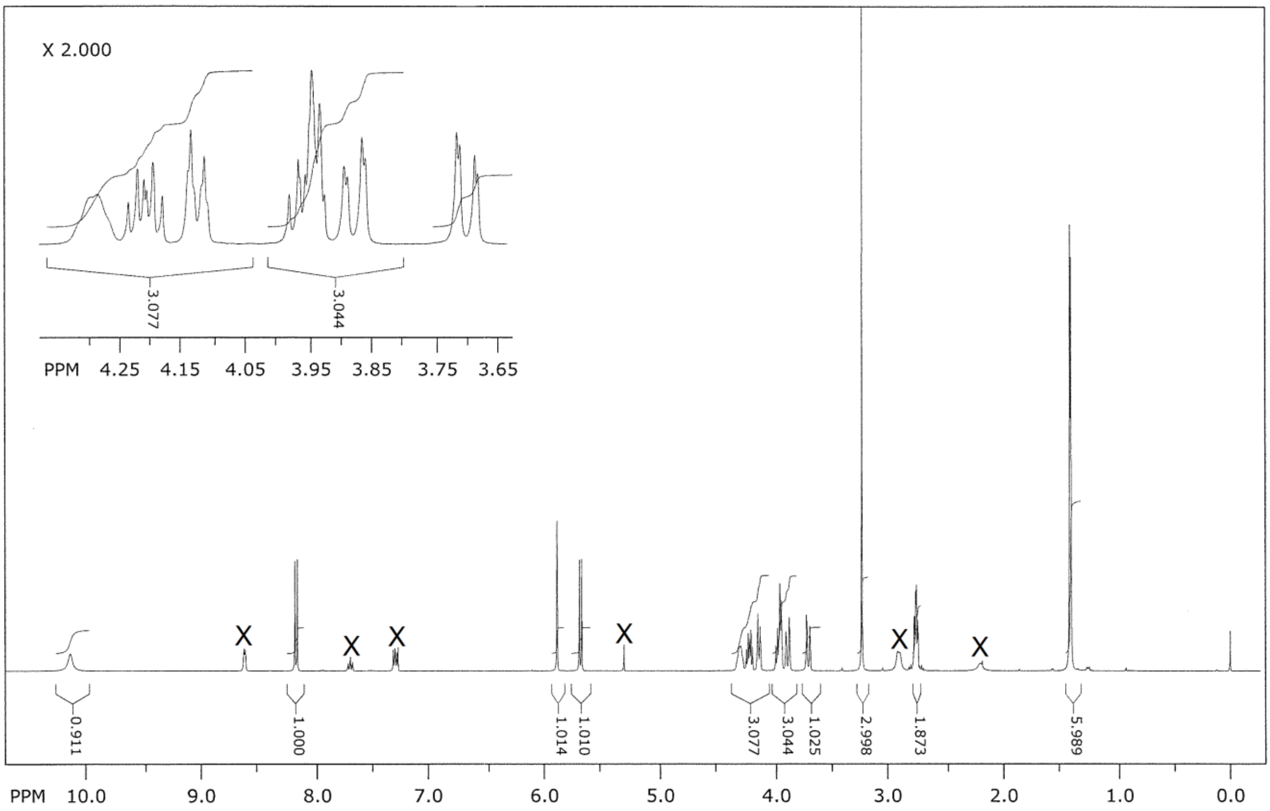

^{13}C NMR. Compound **4a’**

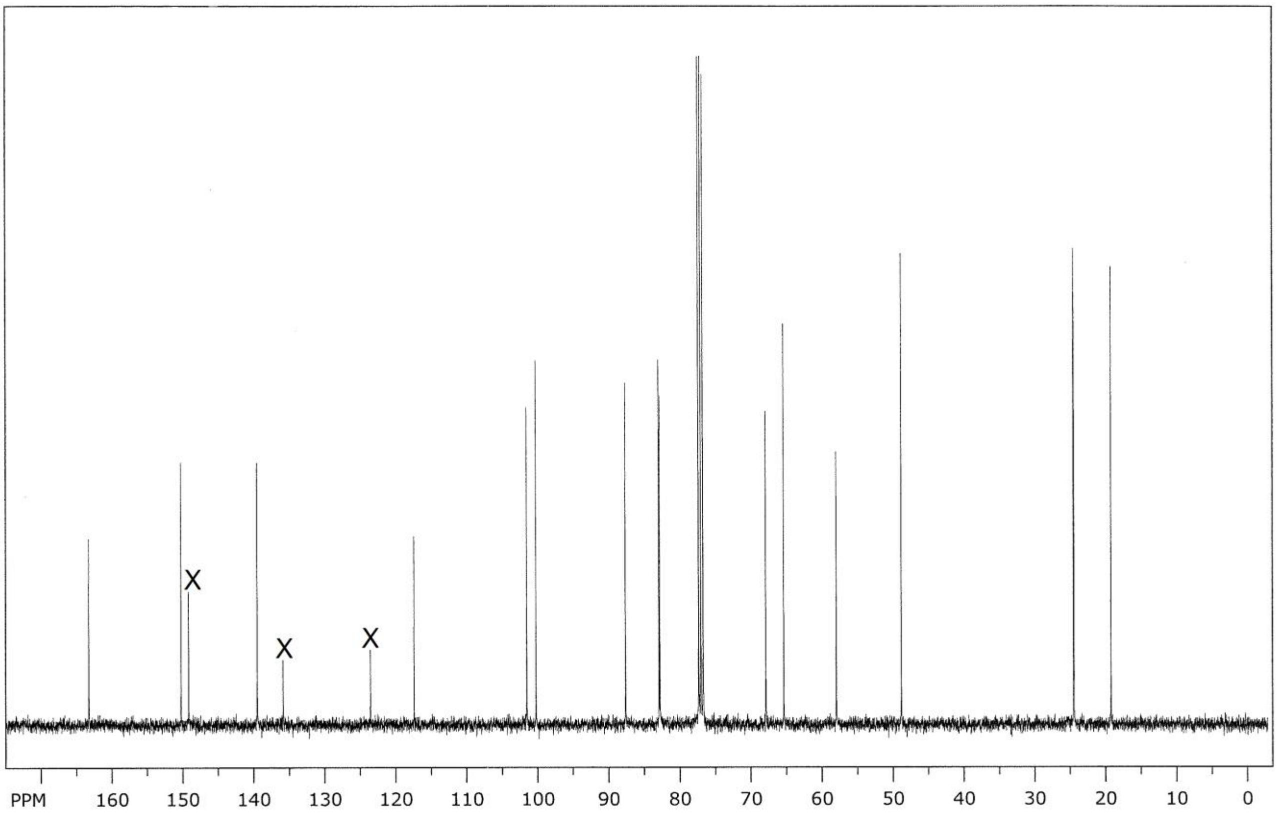

^{1}H NMR. Compound **4b**

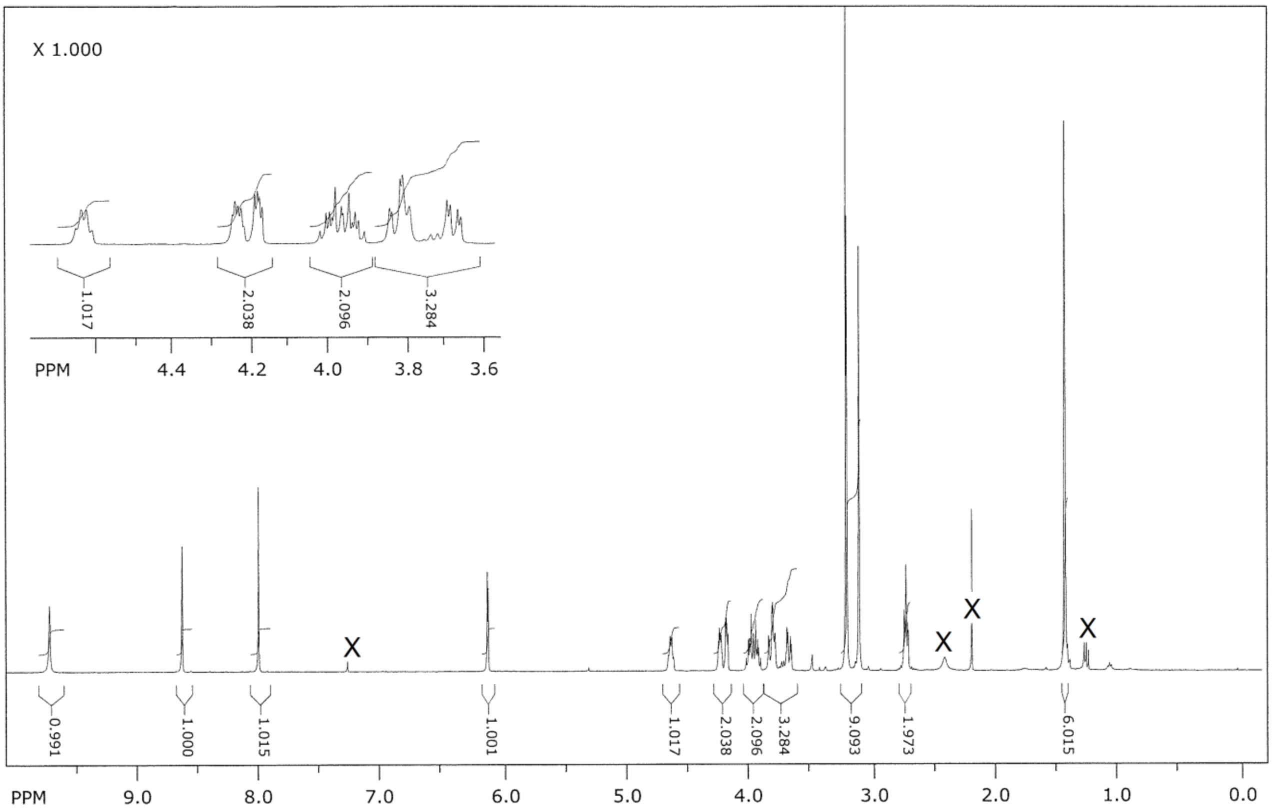

^{13}C NMR. Compound **4b**

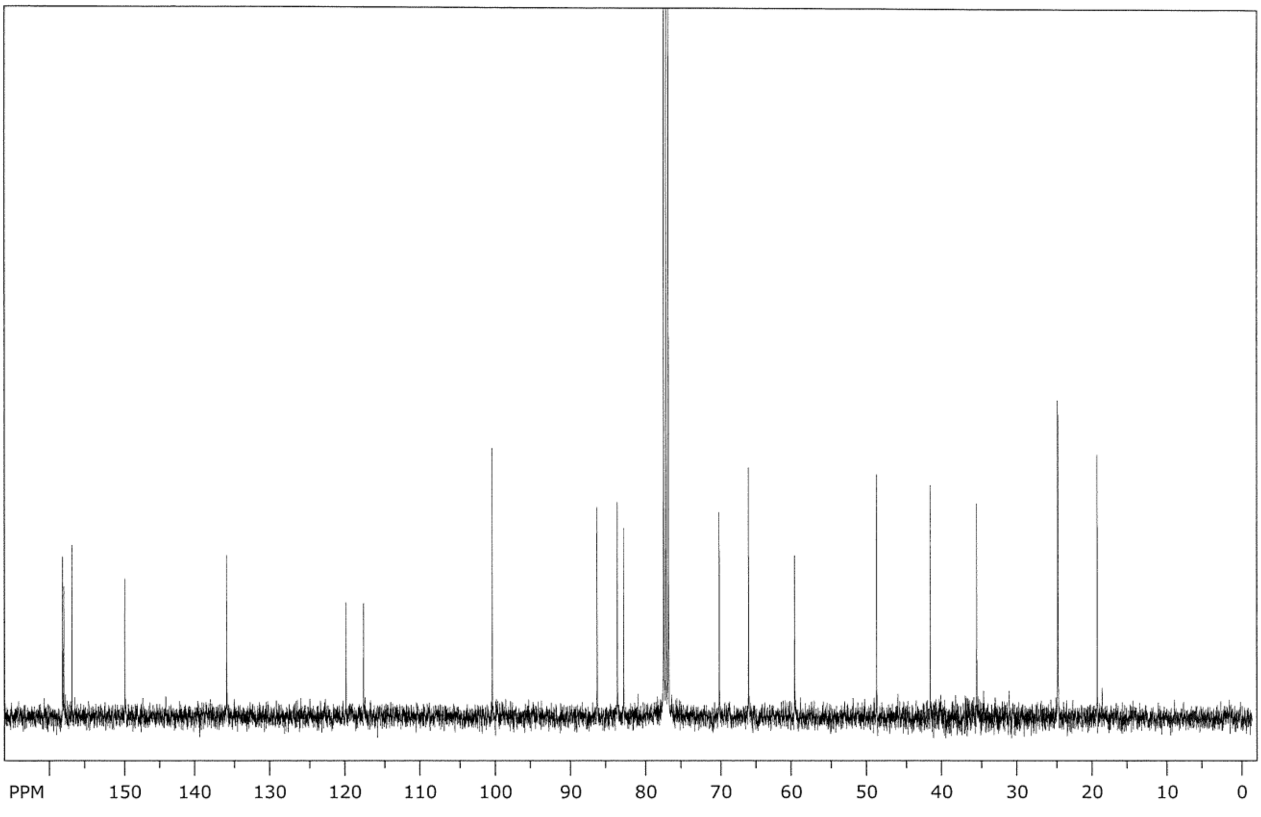

^{1}H NMR. Compound **4c'**

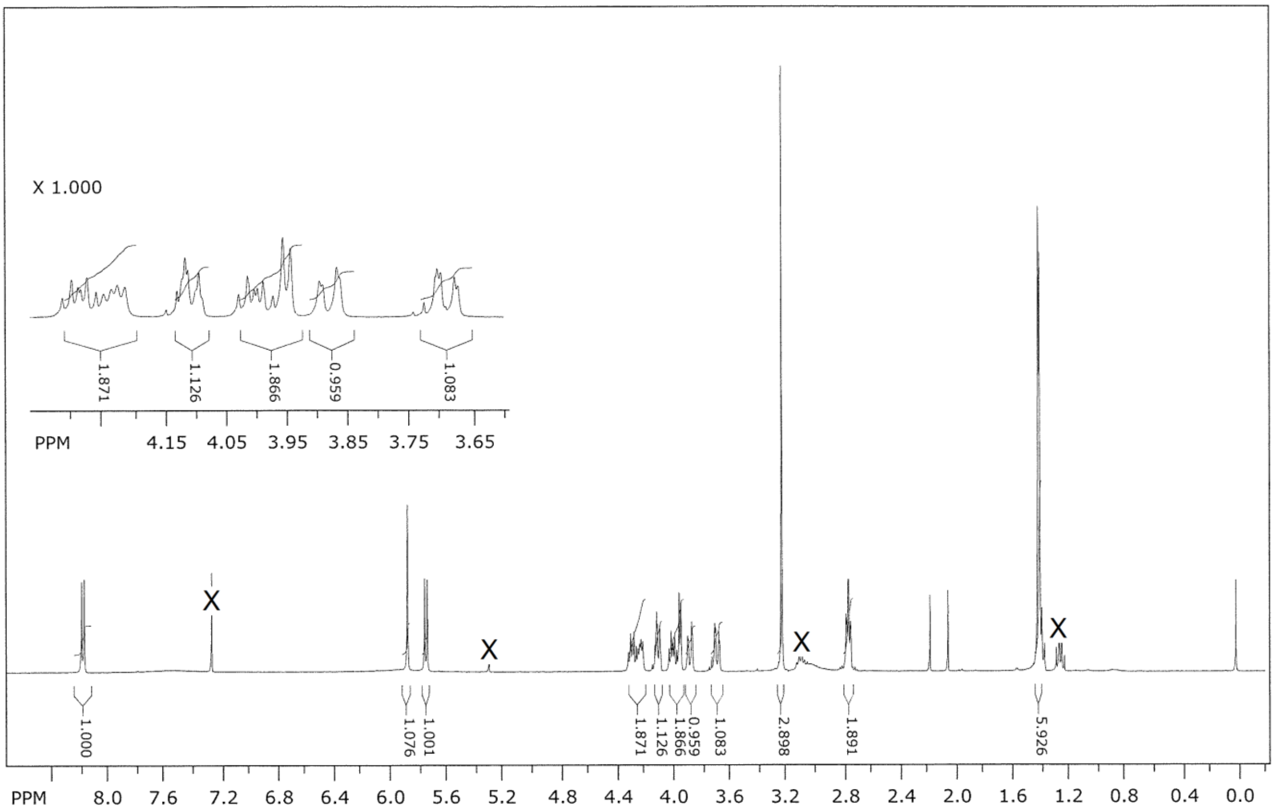

^{13}C NMR. Compound **4c'**

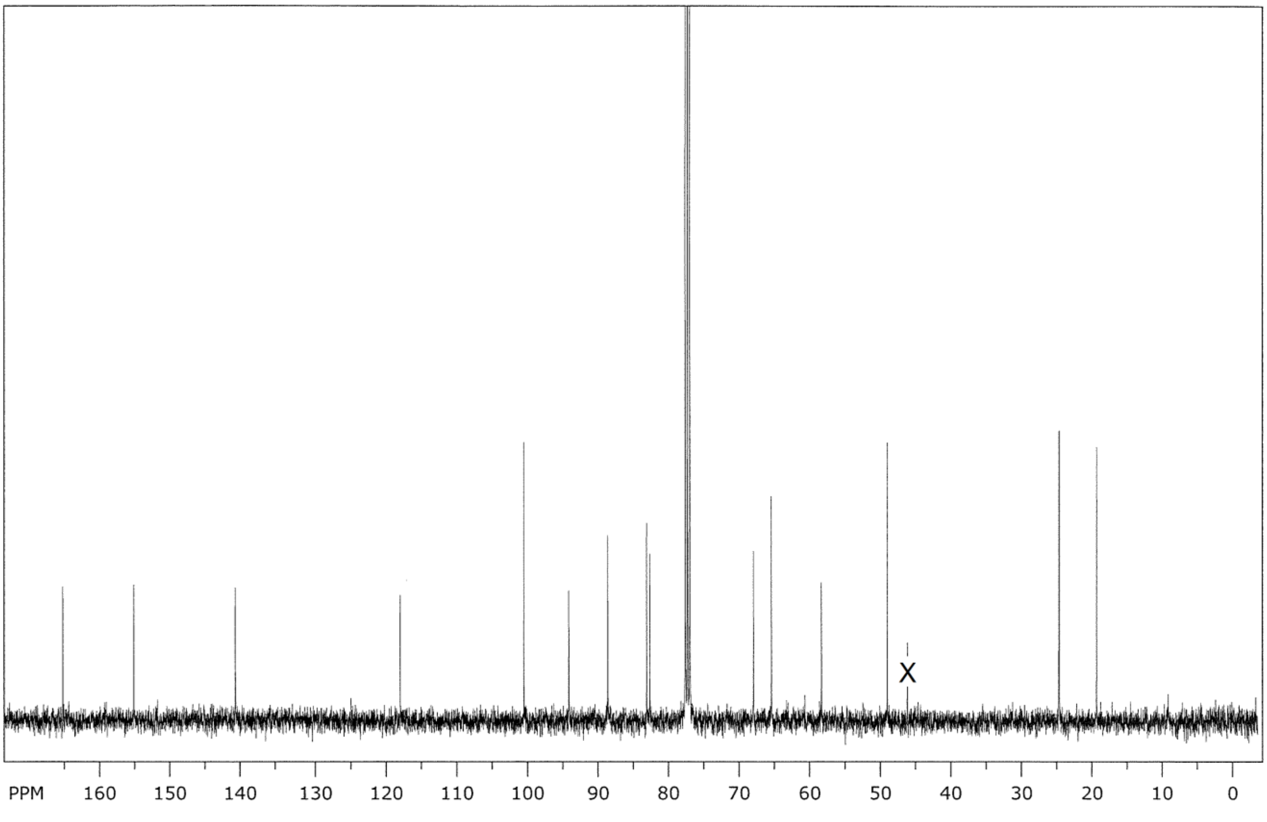

^{1}H NMR. Compound **4c''**

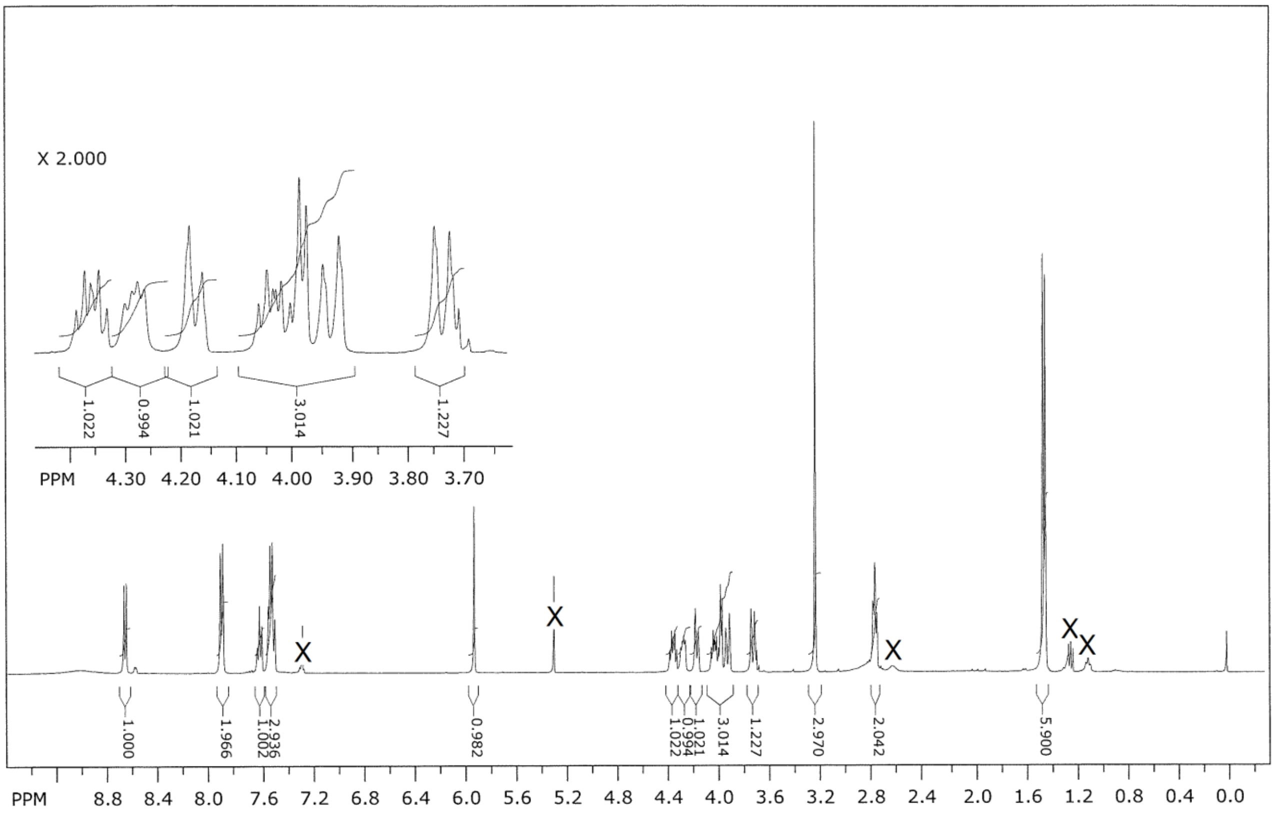

^{13}C NMR. Compound **4c''**

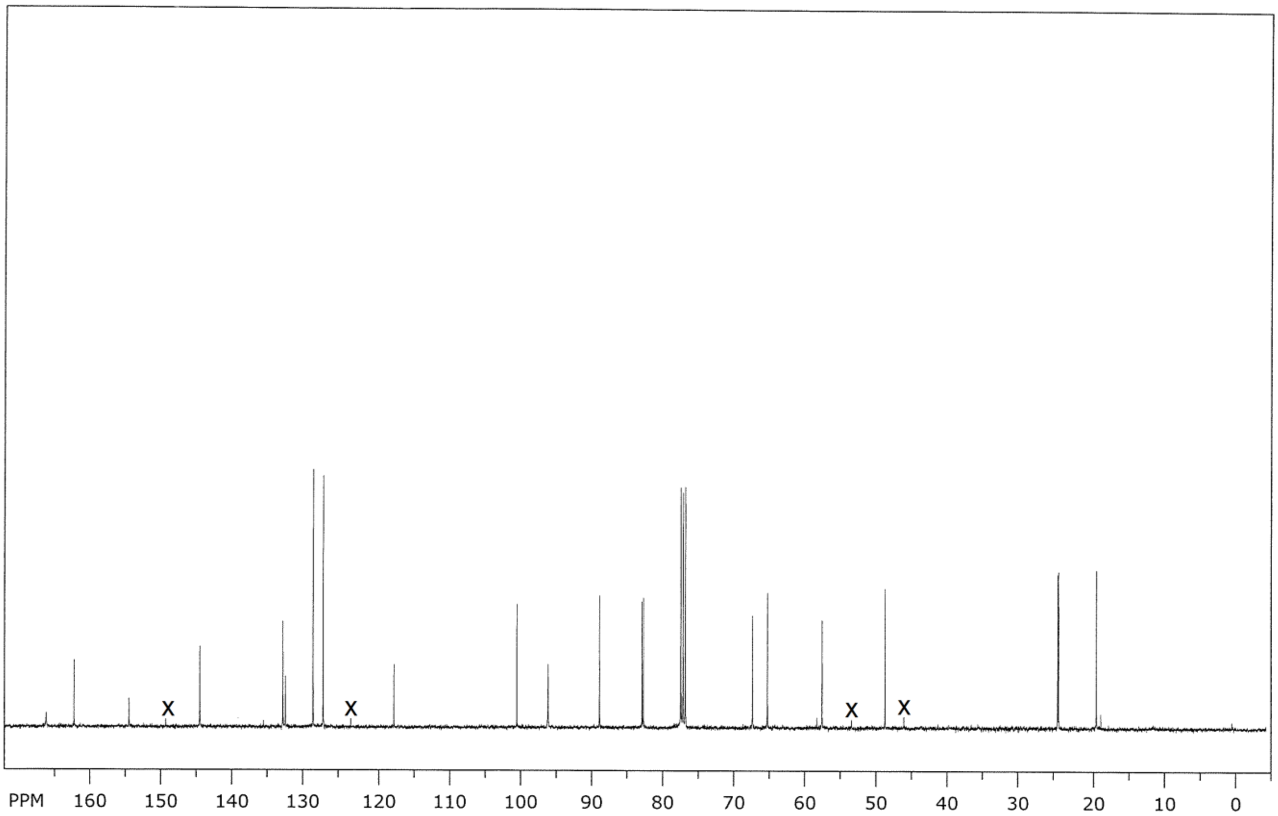

^{1}H NMR. Compound **4d**

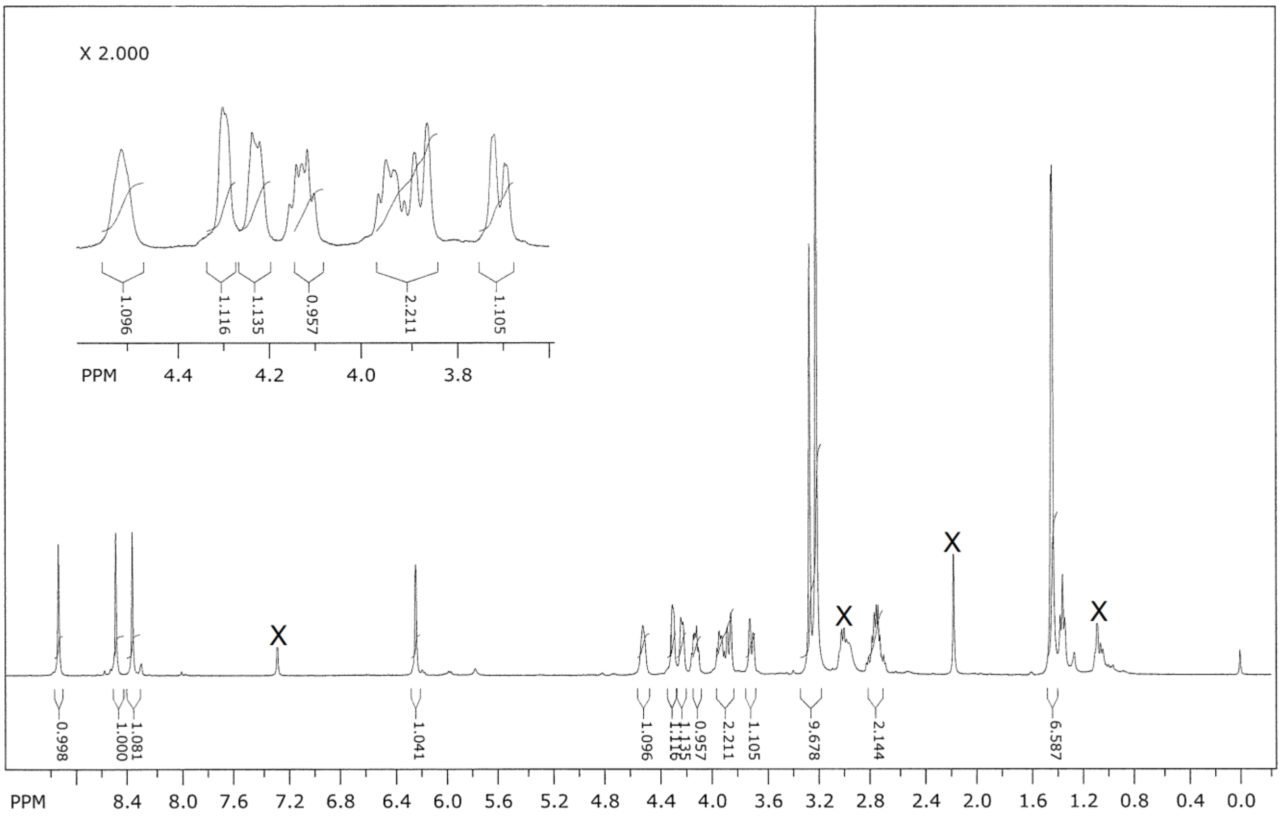

^{13}C NMR. Compound **4d**

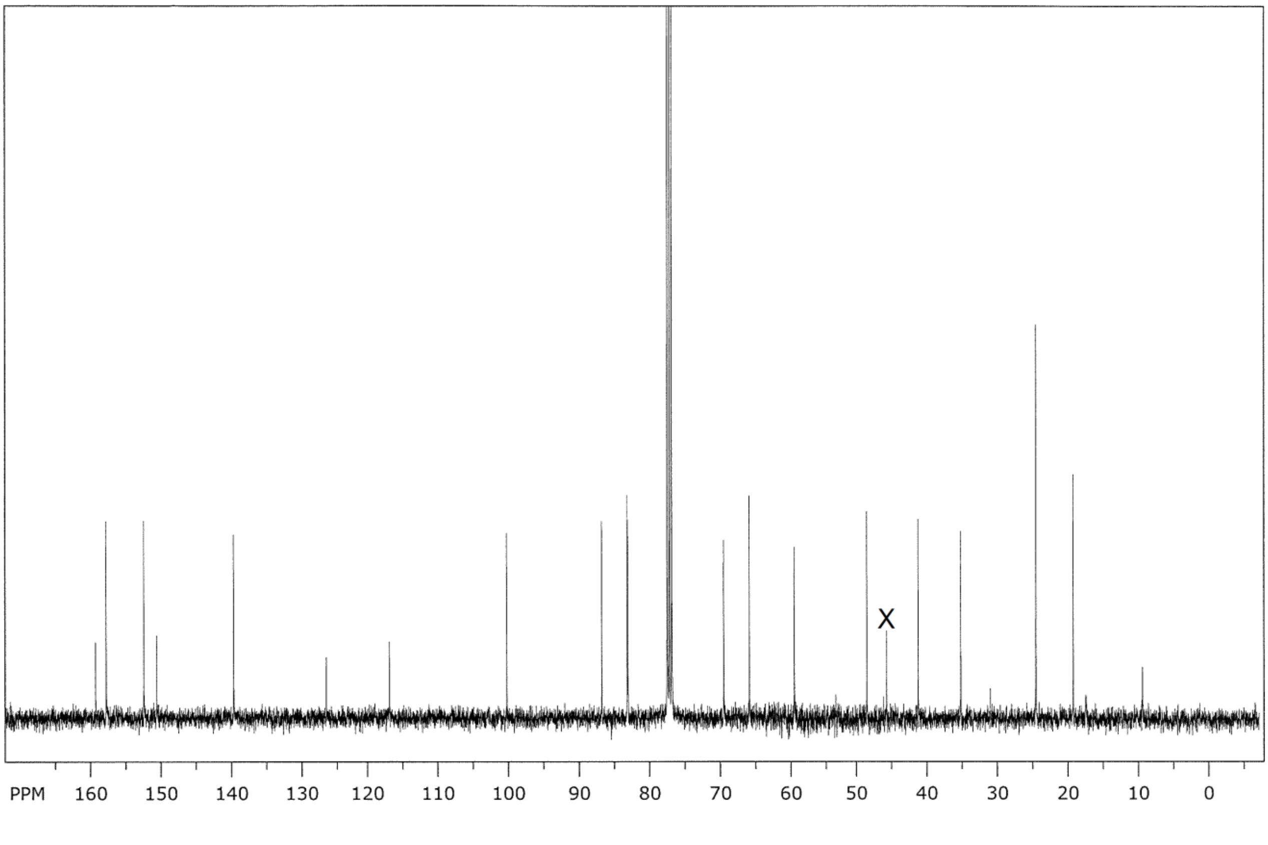

^{31}P NMR. Compound **5a’**

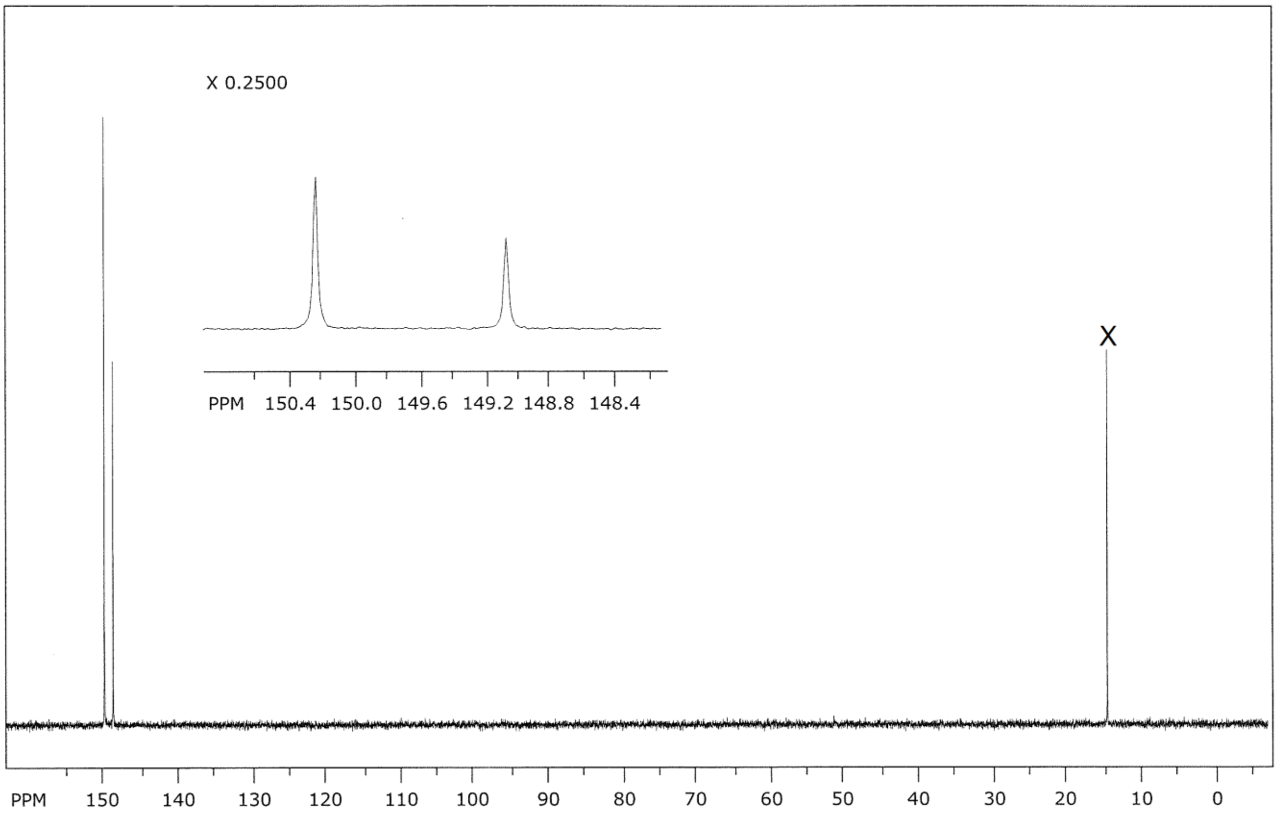

^{31}P NMR. Compound **5b**

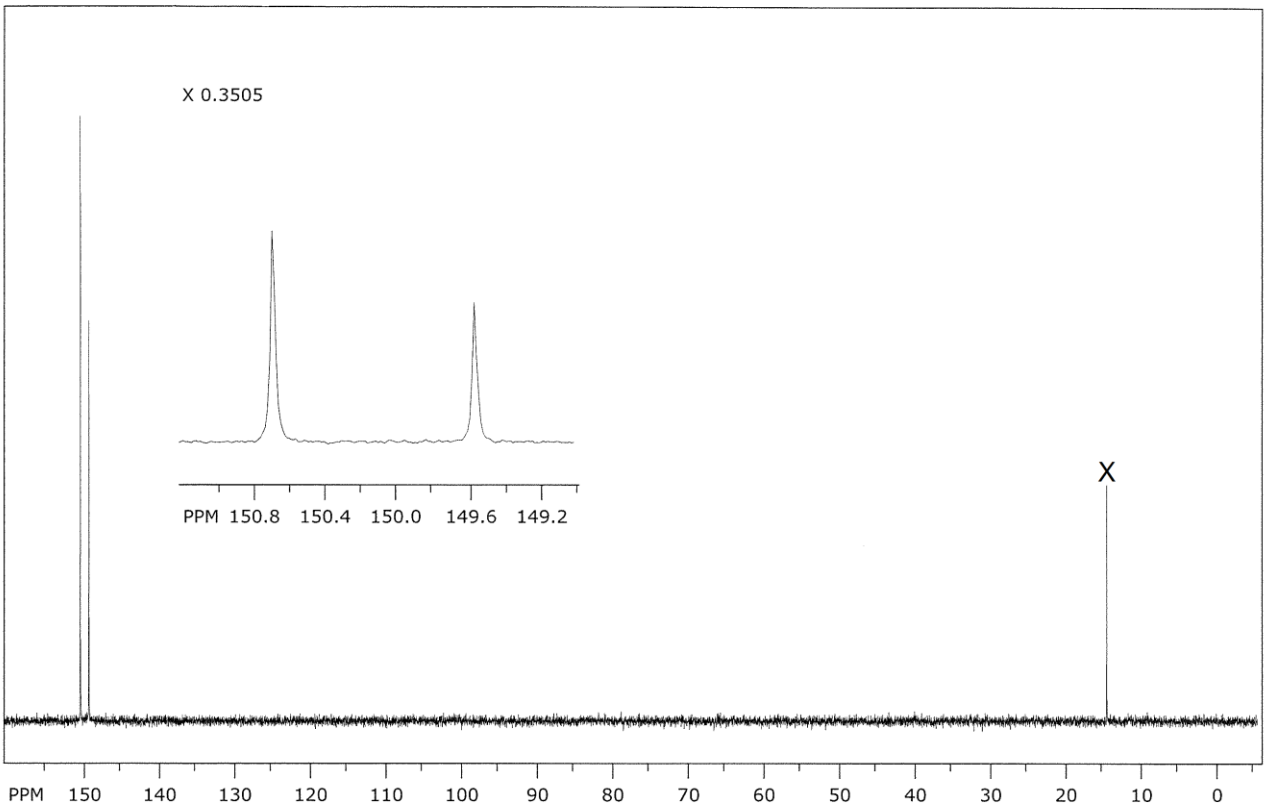

^{31}P NMR. Compound **5c''**

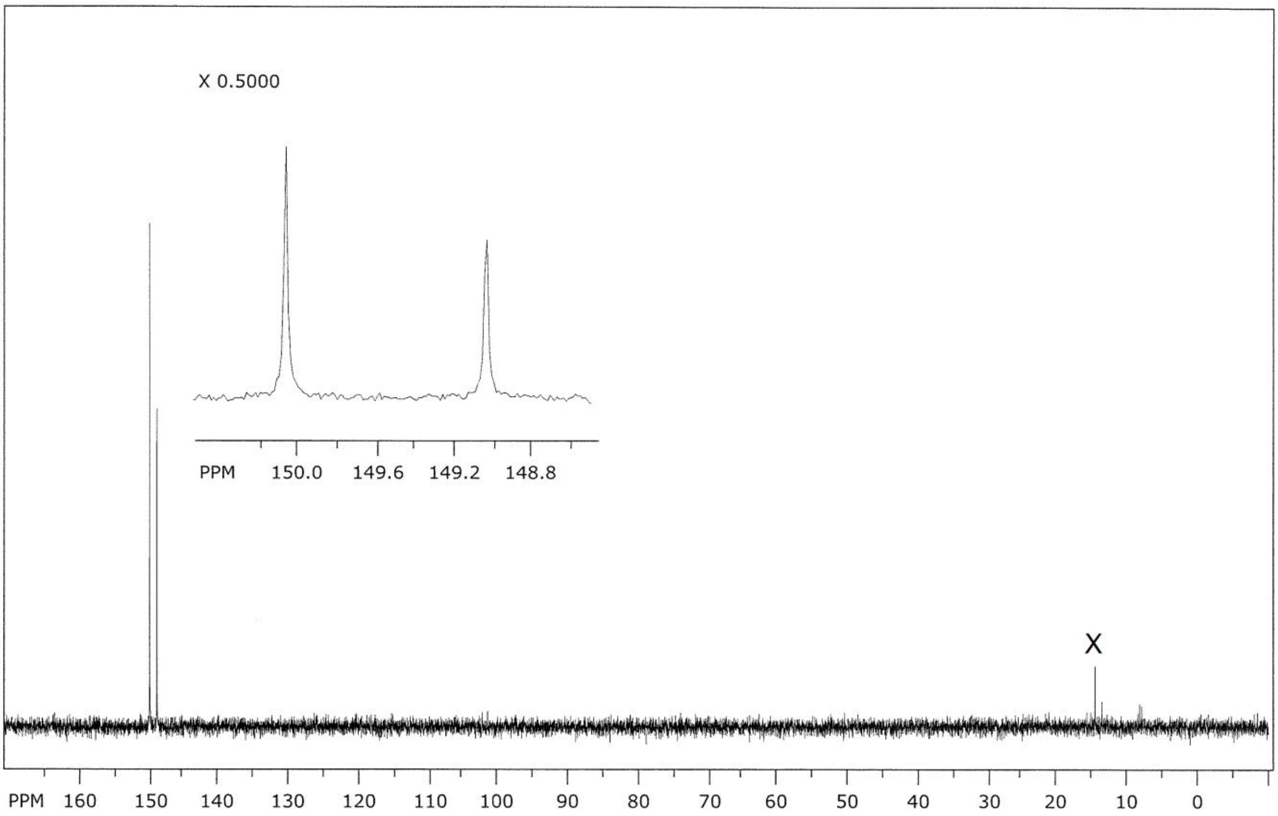

^{31}P NMR. Compound **5d**

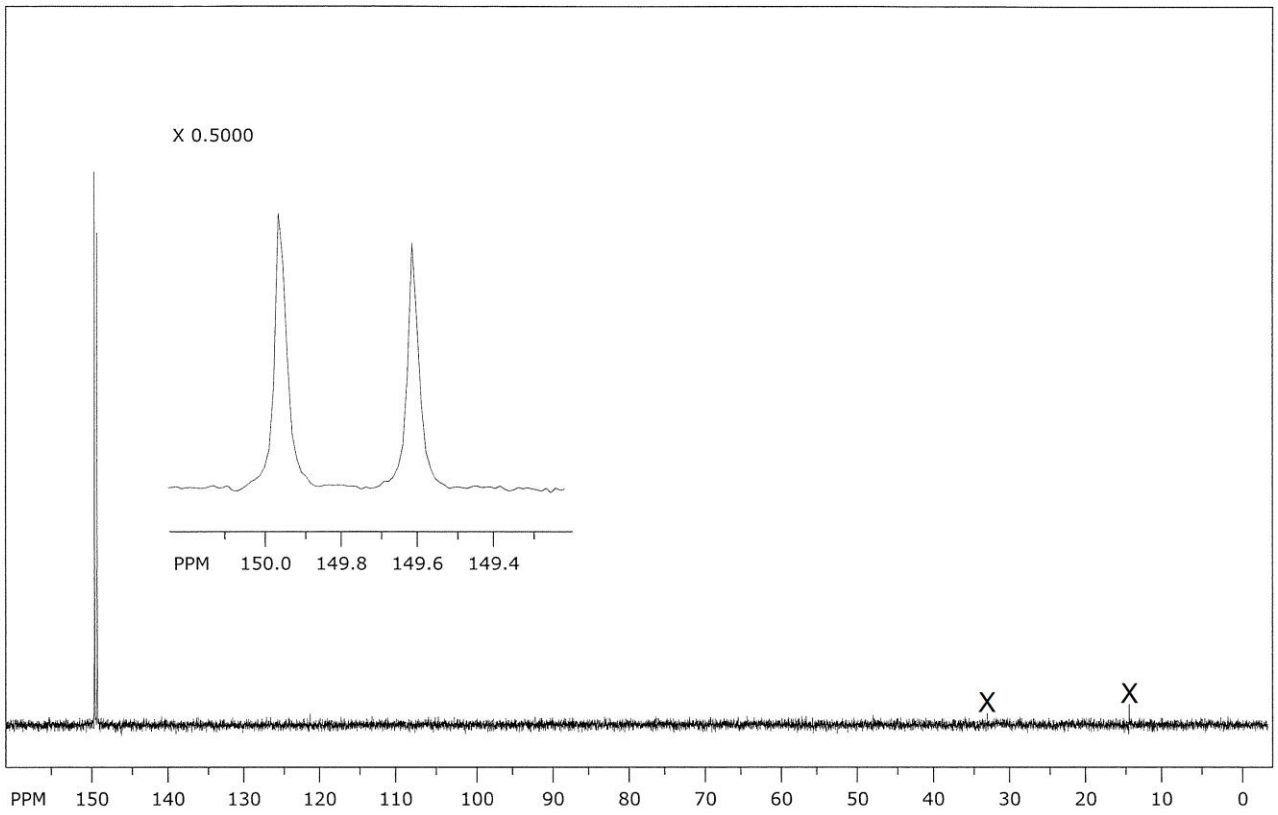

^{1}H NMR. Compound **6a**

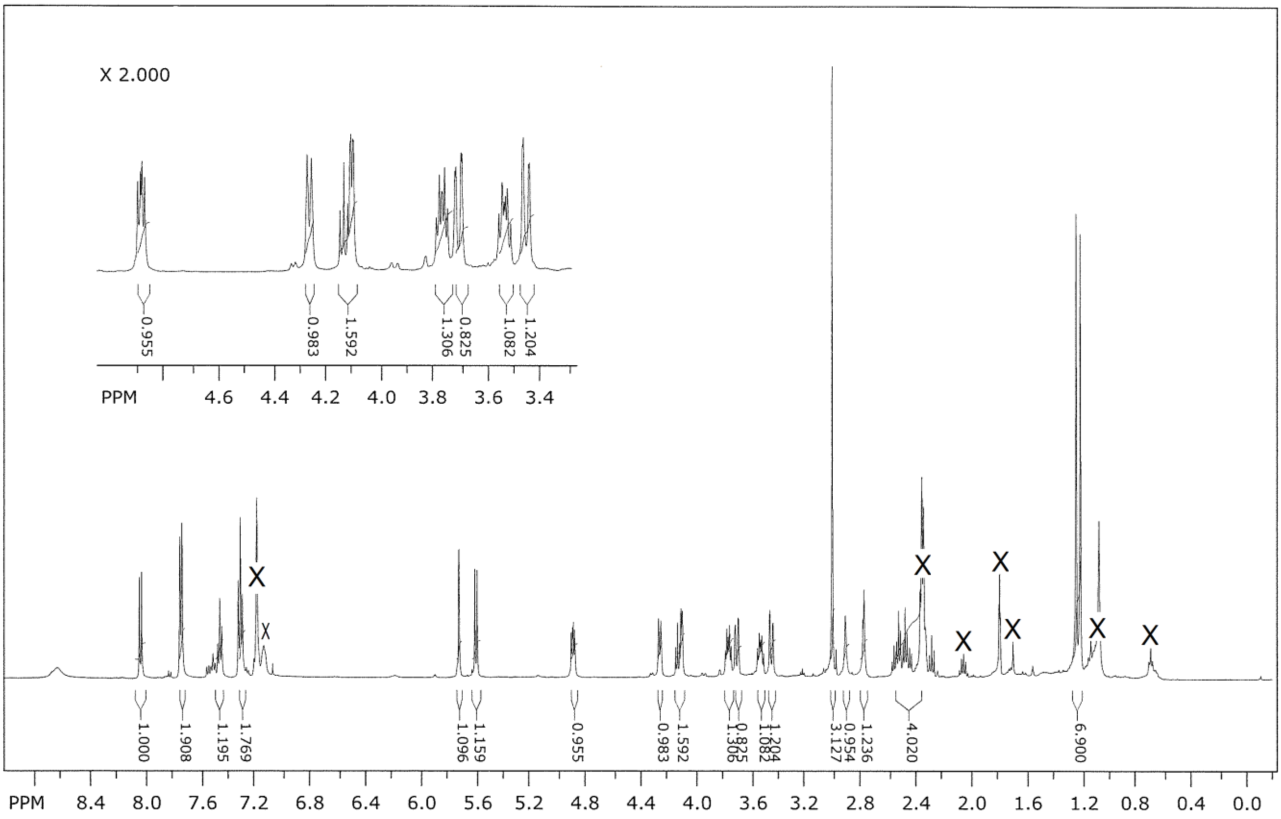

^{13}C NMR. Compound **6a**

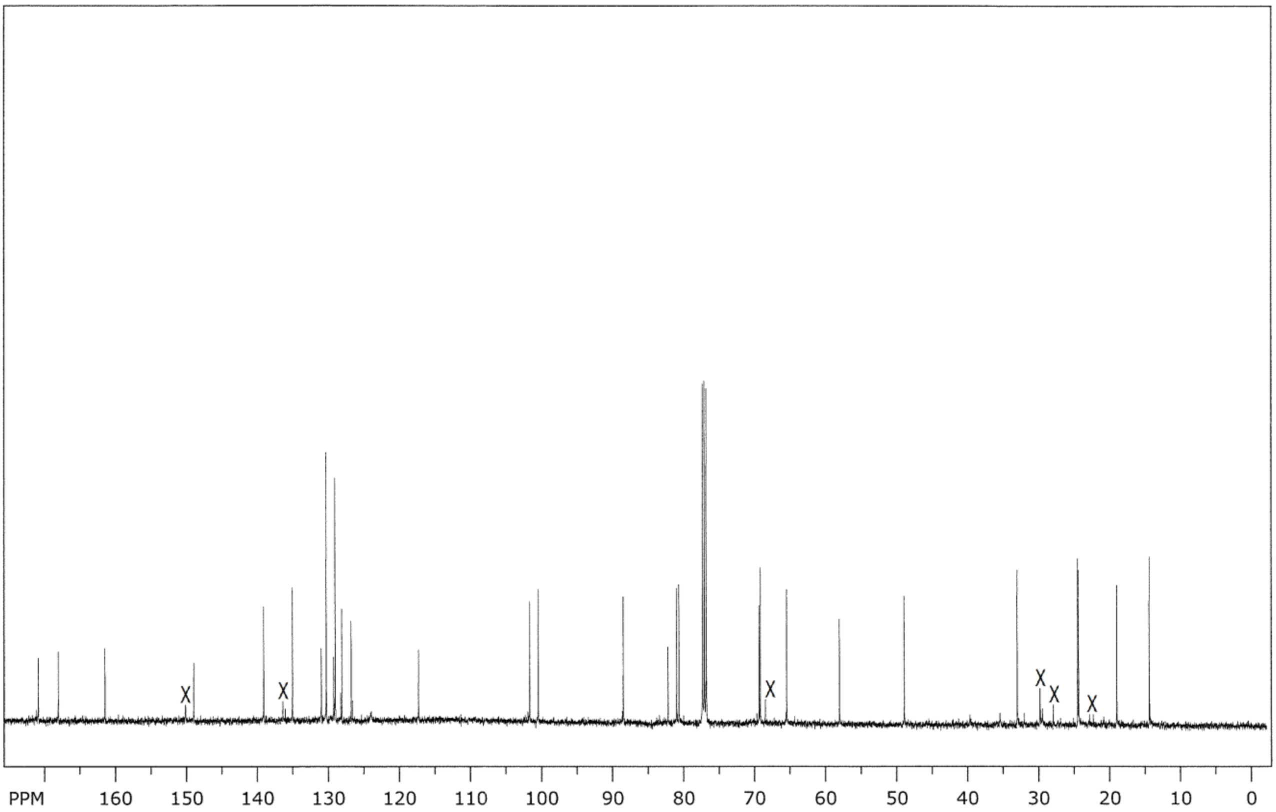

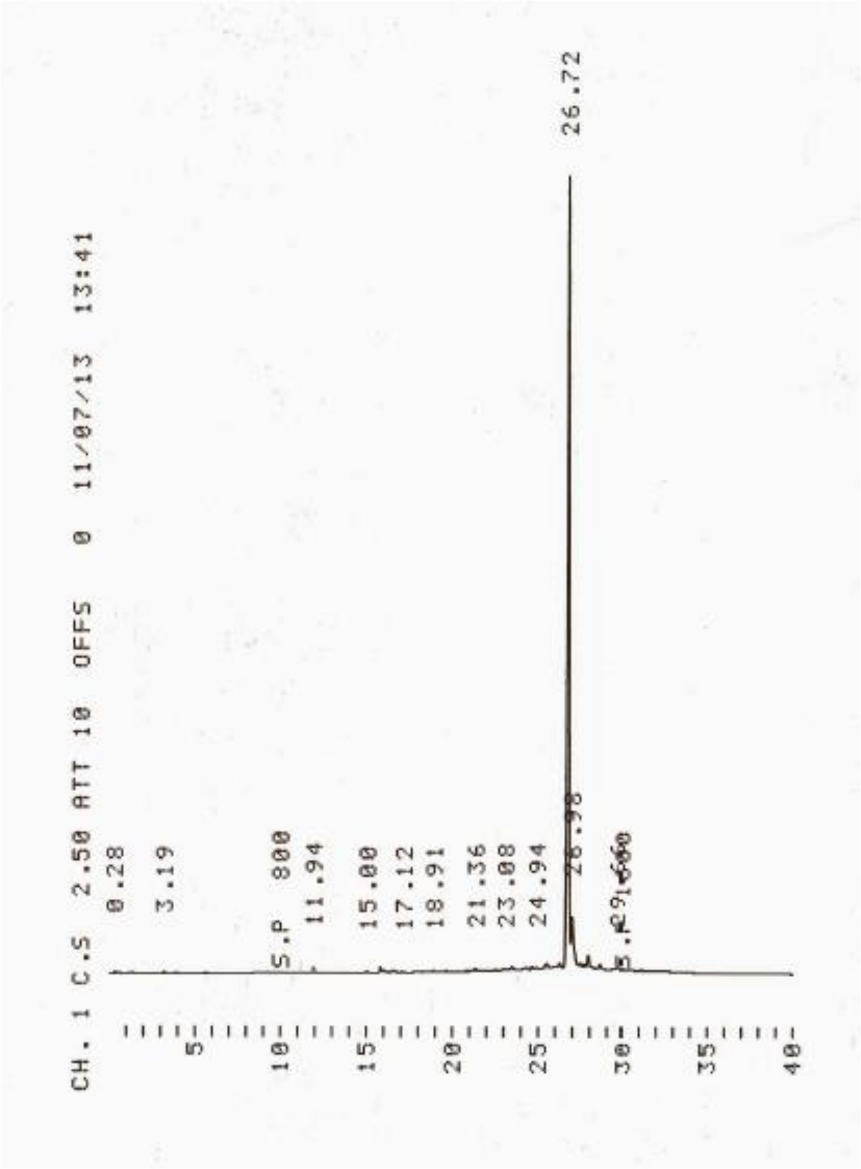

A Thermo ODS Hypersil C18 (250 x 4.6 mm, 5μm) column eluted with a mixture of MeCN and aq Et_3N (0.1 mol L^{-1}) at flow rate 1mL min^{-1}. A linear gradient from MeCN 25% at t = 0 min to MeCN 100% at t = 25min.

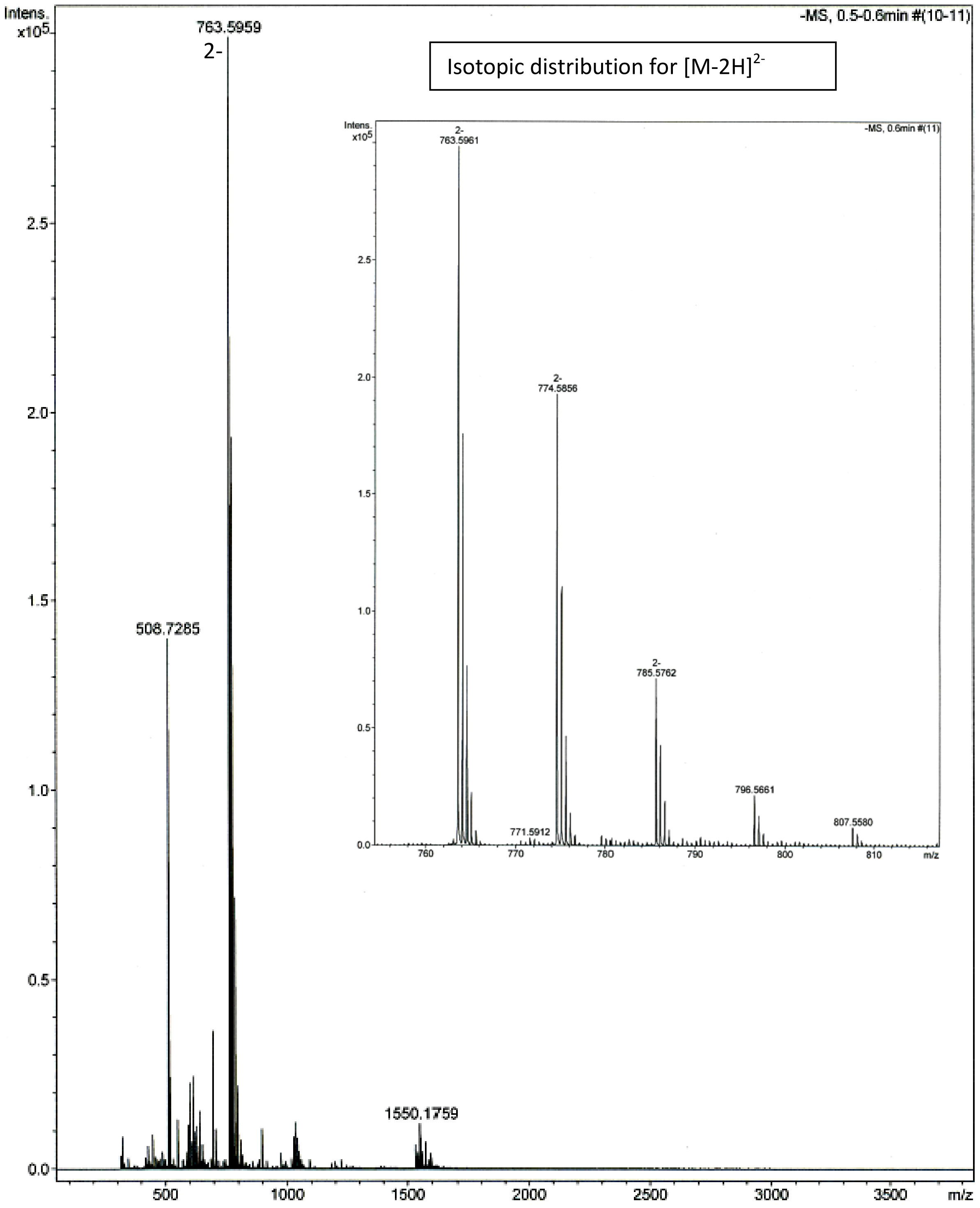

Negative ion ESI-MS of pentamer 3´-UUGCA-5´

References:

[1] Saneyoshi, H.; Seio, K.; Sekine, M. *J. Org. Chem*. **2005**, *70*, 10453-10460.
